# Yippee-like protein Moh1 links gene expression to metabolism and selective stress resistance in *Saccharomyces cerevisiae*

**DOI:** 10.15698/mic2026.06.881

**Published:** 2026-06-29

**Authors:** Çağla Ece Olgun, Gizem Turan Duman, Gizem Güpür, Hamit İzgi, Mariam Huda, Demet Çetin, Zekiye Suludere, Fatma Küçük Baloğlu, Ayşe Koca Çaydaşı, Mesut Muyan

**Affiliations:** 1 Department of Biological Sciences, Middle East Technical University, 06800, Çankaya-Ankara, Türkiye; 2 Department of Molecular Biology and Genetics, Koç University, Istanbul, Türkiye; 3 Department of Mathematics and Science Education, Gazi Faculty of Education, Gazi University, 06500, Ankara, Türkiye; 4 Department of Biology, Faculty of Science, Gazi University, 06500, Ankara, Türkiye; 5 Department of Biology, Giresun University, Giresun, Türkiye

**Keywords:** *MOH1*, *S. cerevisiae*, stress response, SEM, RNA-Seq, FTIR

## Abstract

The Yippee-like (YPEL) proteins are a evolutionarily conserved eukaryotic family implicated in proliferation, senescence, and stress adaptation, yet their molecular functions remain poorly defined. Humans possess five paralogs (YPEL1–YPEL5), while the budding yeast *S. cerevisiae* contains a single ortholog, *MOH1*, previously linked to stress responses but with an unclear cellular role. Here, we investigated the function of *MOH1* in *S. cerevisiae*. *MOH1* deletion resulted in stress-specific phenotypes, including increased sensitivity to sodium azide and sulfuric acid, but enhanced resistance to hydrogen peroxide and acetic acid. Moh1 protein levels were dynamically regulated, decreasing upon hydrogen peroxide treatment and increasing in response to sulfuric acid. Morphological analyses including SEM revealed that *moh1* Δ cells are rounder, form aggregates, and exhibit altered surface architecture independently of stress. RNA profiling and FTIR spectroscopy uncovered transcriptional reprogramming and metabolic remodeling, including alterations in lipid, protein, and cell wall polysaccharide levels and composition. Functional analyses showed that increased resistance to hydrogen peroxide is not due to altered mitochondrial ROS production but rather to reduced intracellular ROS accumulation. This effect is attributed to decreased cellular uptake resulting from altered permeability, supported by resistance to Congo red and sensitivity to SDS, consistent with cell envelope remodeling. Collectively, our findings identify Moh1 as a regulatory factor linking gene expression to metabolism and cellular architecture, thereby influencing cell envelope permeability and conferring selective stress resistance in *S. cerevisiae*.

## INTRODUCTION

The *YPEL* family has 100 genes in 68 species, ranging from yeast and plants to mammals, with a remarkably high nucleotide sequence identity 1–3. While the budding yeast *Saccharomyces cerevisiae* (*S. cerevisiae*) contains a single gene named MOH1 (YBL049W), the human *YPEL* family includes five YPEL genes, *YPEL1-5*, located on different chromosomes, which are suggested to have arisen from ancestral gene duplications 1–3. The YPEL2, YPEL3, and YPEL5 genes are ubiquitously expressed, albeit at varying levels, in human tissues; whereas, *YPEL1* and *YPEL4* show expression patterns largely restricted to the testis and brain, respectively 2. In addition to the ubiquitous tissue expressions, various signaling pathways, including 17 β -estradiol (E2)-estrogen receptor (ER) signaling 4, 5, co-modulate the expressions of *YPEL2, YPEL3*, and *YPEL5*.

The YPEL family genes encode small proteins with a high amino-acid sequence identity predicted to form a zinc-finger-like metal binding pocket, or the Yippee domain 1, 2. This high degree of sequence identity among YPEL proteins results in structural conservation that is likely reflected in functional commonalities, as YPEL proteins are involved in similar cellular processes, ranging from proliferation, senescence, and death 3, 4, 6–22. Consistent with these, deregulated YPEL gene expressions have been suggested to be associated with the initiation and/or development of various disorders, malignancies, and resistance to therapies 6, 12, 13, 20, 23–29.

**Figure 1:**
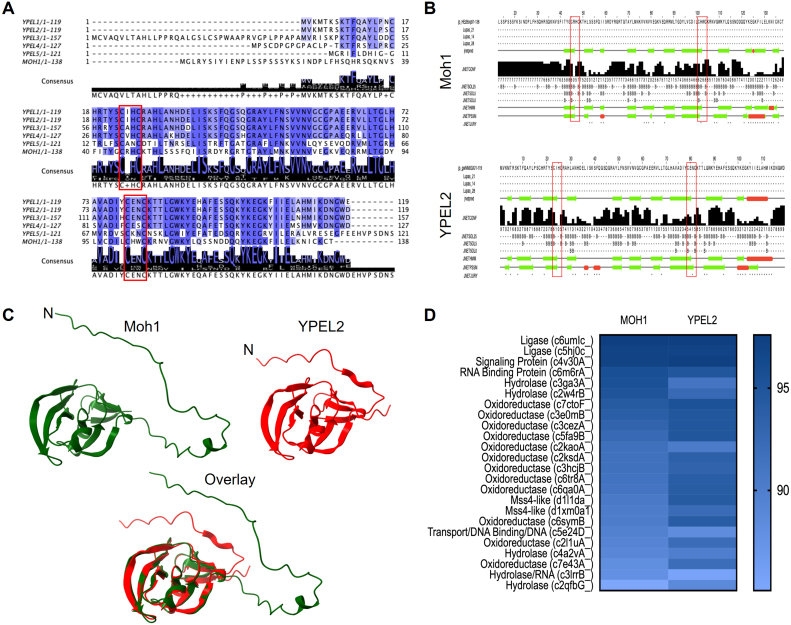
*In silico* analyses of Moh1 and the YPEL family proteins . **(A)** The alignment of the amino acid sequence of YPEL proteins and Moh1. Red squares indicate CXXC motifs. **(B)** Secondary structure analysis of Moh1 and YPEL2 with the jPred server **(C)**. Prediction and superimposition of tertiary structures of YPEL2 and Moh1 with the AlphaFold server using the ChimeraX molecular visualization program. **(D)** The Phyre2 web tool was used for the homology modeling of Moh1 and YPEL2.

Despite the potential importance of YPEL proteins in physiology and pathophysiology, the evolutionary conservation in nucleotide sequences, the ubiquity, and commonalities in the regulation of YPEL gene expressions, as well as structural similarities among YPEL proteins, render the deciphering of functional features of a YPEL protein in cellular processes in the presence of other YPELs difficult. We recently employed an inducible heterologous expression system, combined with dynamic proximity biotin labeling and mass spectrometry analyses, in non-tumorogenic COS7 cells, an immortalized African green monkey kidney fibroblast-like cell line that synthesizes endogenous YPEL protein(s). Results indicated that YPEL2 interacts with proteins involved in cellular processes, including stress response 30. Based on these and our observations that YPEL2, as the endogenous YPEL protein(s), localizes to stress granules in response to oxidative stress, we suggested that YPEL2 participates in stress surveillance 30. However, the mechanism(s) by which YPEL2 exerts its effects on cells remain unclear.

**Figure 2:**
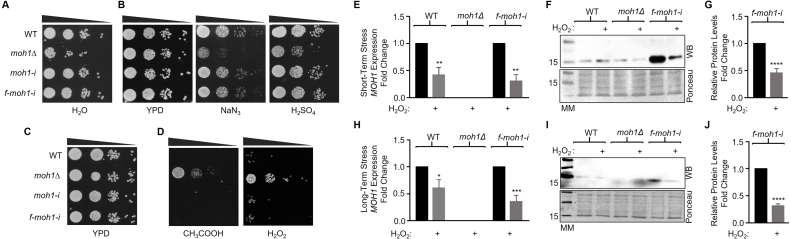
Spot tests of yeast strains grown on agar plates without or with a stressor . **(A)** A single colony from WT, *moh1* Δ , *moh1-i*, and *f-moh1-i* strains was grown in YPD overnight. 50 × 10 ${}^{6}$ cells were subcultured into 20 mL of YPD at a ratio of 1:100 and grown for one week. Cells were then washed twice with 1 M Sorbitol and once with sterile distilled water, and resuspended in 20 mL of sterile distilled water. Cells were incubated at 30 ${}^{\circ }$ C with shaking at 180 rpm. Cultures were collected for a spot test at day 14 and spotted on agar plates with 10-fold serial dilutions (Black triangles). Cells were incubated at 30 ${}^{\circ }$ C for 40 hours and photographed. **(B–D)** Cells from subcultures were grown until OD ${}_{600}$ 0.4–0.6. Cells, 2.5 × 10 ${}^{6}$ cells/mL, were spotted with 10-fold serial dilutions on **(B)** YPD-Agar plates containing none (YPD) or 0.4 mM NaN3, or 0.22% H ${}_{2}$ SO ${}_{4}$ , **(C)** none (YPD), **(D)** 40 mM CH ${}_{3}$ COOH, or 3.25 mM H ${}_{2}$ O ${}_{2}$ . Plates were incubated at 30 ${}^{\circ }$ C for 40 hours and photographed. **(E–J)** Expression by RT-qPCR **(E & H)** and synthesis by WB using the Flag antibody **(F & I)** of *MOH1* in WT, *moh1* Δ , and *f-moh1-i* cells after exposure to short-term, 45 min **(F & G)** or long-term stress, 40 h. **(I & J)** Expression of *MOH1* using primers specific to *MOH1* was normalized to transcript levels of *YPR062W* (*FCY1*) and *YNL219C* (*ALG9*) as internal controls in RT-qPCR. In WB analysis, the level of Flag-Moh1 migrating at ∼ 16 kDa in the absence of H ${}_{2}$ O ${}_{2}$ was set to one and compared to that observed in the presence of H ${}_{2}$ O ${}_{2}$ . A band of similar molecular mass was also detected in both WT and *moh1* Δ strains, indicating that it represents a non-specific signal. Ponceau staining was used as a control for equal protein loading. Molecular weight markers are indicated in kDa. A Student’s t-test was conducted for statistical analyses. ${}^{*}$ , ${}^{**}$ , ${}^{***}$ , and ${}^{****}$ indicate p < 0.05, p < 0.01, p < 0.001, and p < 0.0001, respectively.

The basic mechanisms of cellular events, including proliferation, metabolism, and death, in the budding yeast *S. cerevisiae* share characteristics with those of evolutionarily distant species, such as humans, due to their highly conserved gene homology 31. This allows the implementation of functional complementation approaches to address the features of homologous proteins. Since the yeast MOH1 (YBL049W) gene is the ortholog of the human YPEL gene family, we envisioned that complementing *MOH1* in a yeast model with a human YPEL gene could yield significant information about the role of a YPEL protein in cellular processes independently of other YPEL proteins.

A limited number of studies suggest that the MOH1 gene is a stationary phase-essential gene 32, as *MOH1*-deleted (*moh1* Δ ) cells cannot survive under nutrient-depleted stationary phase conditions 32, 33. This is consistent with observations that the exit from the stationary phase in response to a nutrient-rich environment augments a set of gene expressions, including *MOH1* 34. Moreover, various stressors, including heat, zinc ion starvation, and hydrogen peroxide, modulate *MOH1* expression 35–38. Extending these observations, *moh1* Δ cells have been reported to exhibit enhanced cell viability compared to the wild-type (WT) strain when treated with various stressors, including UV irradiation, chemicals, heat, and hyperosmotic shock 22. Furthermore, the ectopic expression of *MOH1* or individual YPEL genes in the *moh1* Δ strain restores the WT phenotype in response to stressors 22. In addition, recent studies reported that the genotoxic agent methyl methanesulfonate induces *MOH1* expression 39 and that *MOH1* may act as a stress response regulator, enhancing sensitivity to DNA damage in *Candida albicans* 40. These studies collectively suggest that Moh1p, like the human YPEL2 protein, is involved in cell survival and stress response. However, how Moh1p exerts its effect on cellular phenotype is unclear. Since the elucidation of functional features of Moh1p could provide important clues about YPEL functions, here we used *S. cerevisiae* as a model system to initially explore the effects of *MOH1* on molecular events. Our findings, obtained using light and scanning electron microscopy (SEM), RNA sequencing (RNA-Seq), and Fourier Transform-Infrared Spectroscopy (FTIR), indicate that the deletion of *MOH1* in *S. cerevisiae* leads to constitutive molecular alterations that affect cell wall integrity, conferring selective advantages/sensitivities under specific stress conditions. Our results suggest that Moh1p constitutively influences metabolic and physiological processes critical for selective stress adaptation and cell survival.

## RESULTS

### Structural analysis of Moh1p and its similarity to YPEL proteins

Although the YPEL protein family is structurally conserved, most analyses have focused on primary sequence. Alignments based on the highly conserved cysteine residues reveal a striking amino acid identity among YPEL proteins, with numerous identical residues forming the Yippee domain. This domain, which contains two cysteine pairs separated by 52 amino acids (Cys-X ${}_{2}$ -Cys-X ${}_{52}$ -Cys-X ${}_{2}$ -Cys), is predicted to form a zinc-binding pocket 1, 2 critical for folding and generating a presumably ligand-binding aromatic cage 41, 42.

To assess the relationship between yeast Moh1p and human YPEL proteins, we performed *in silico* analyses. Multiple sequence alignment (Clustal Omega, visualized with Jalview) 43, 44 confirmed that human YPEL1–4 share ∼ 83% identity, while YPEL5 is less similar (43.8–49.5%) (Figure 1**A**) 1, 2. *S. cerevisiae* Moh1p shares 37.8% identity with YPEL2 and 31.4–40.2% with other YPEL proteins. Secondary structure predictions using the JPred4 server 45 indicated that both Moh1p and YPEL2 are rich in β -strands (Figure 1**B**). Tertiary structure models using AlphaFold and visualization with ChimeraX 46–49 further showed that Moh1p and YPEL2 adopt similar globular, superimposable folds, differing mainly at their amino-termini (Figure 1**C**). Additional structural comparisons using Phyre2 50 predicted high-confidence structural matches (>90%), suggesting that the Yippee domain is a conserved fold found across a broad range of functionally diverse protein families, including ligases, RNA-binding proteins, hydrolases, oxidoreductases, and Mss4-like proteins involved in essential cellular processes such as proteolysis, centromere priming, oxidative stress defense, and stress responses (Figure 1**D**, Supplementary Information Table S1) 51–59. However, the functional role of the Yippee domain in these proteins remains largely unclear. Nonetheless, *MOH1* has been reported to be essential for survival in the stationary phase, as *moh1* Δ cells are unable to proliferate under nutrient-depleted, respiration-dependent conditions 32, 33, 60. Consistent with this, the expression of *MOH1* was found to increase upon re-entry into growth from the stationary phase 34. Additionally, *moh1* Δ cells exhibit altered viability under UV, heat, chemicals, and osmotic shock, which can be rescued by ectopic MOH1 or a YPEL gene expression 22. These findings, together with our recent observations that YPEL2 is involved in oxidative stress surveillance 30, suggest a role for Moh1p in stress response.

### Effects of *MOH1* deletion on cell survival under various stress conditions

Since *moh1* Δ cells cannot grow under nutrient-depleted, respiration-dependent conditions 33, 61, we aimed to validate this observation by comparing the colony-forming ability of WT and *moh1* Δ cells after 18 days in water, which mimics nutrient-depleted conditions (Figure 2**A**). We also included the *MOH1*-rescued strains in which *MOH1* (*moh1-i*) or a Flag-tagged *MOH1* (*f-moh1-i*) was reintroduced into the *moh1* Δ background to determine whether the insertion of *MOH1* rescues the phenotype observed in *moh1* Δ cells (Figure 2**A**). Consistent with earlier findings 32, 33, *moh1* Δ cells showed reduced survival compared to WT cells, while both rescued strains restored growth (Figure 2**A**). Importantly, the Flag tag did not impair Moh1p function, confirming that the *f-moh1-i* strain is suitable for protein-level analyses in our subsequent experiments.

Unlike the reduced ability of *moh1* Δ cells to resume growth after the stationary phase, *moh1* Δ cells were reported to grow comparatively better than WT cells following DNA damage, heat shock, and hyperosmotic shock treatments 22. To further examine the growth and survival of yeast cells in response to various stress conditions, we subjected the WT and *moh1* Δ strains to 0.4 mM sodium azide (NaN ${}_{3}$ , a cytochrome oxidase blocker for the mitochondrial respiratory chain), 40 mM acetic acid (CH ${}_{3}$ COOH, a weak organic acid), 0.22% sulfuric acid (H ${}_{2}$ SO ${}_{4}$ , a strong acid), and 3.25 mM hydrogen peroxide (H ${}_{2}$ O ${}_{2}$ , an oxidative stress inducer) 62–66. Briefly, strains cultured in YPD medium to the logarithmic phase were spotted on YPD agar plates containing each stressor. Stressor concentrations were chosen empirically to produce measurable but non-lethal effects (Supplementary Information Fig. S1). Spot assays showed that in the presence of NaN ${}_{3}$ or H ${}_{2}$ SO ${}_{4}$ , WT cells showed better survival compared to *moh1* Δ cells (Figure 2**B**). In contrast, the *moh1* Δ cells were more resistant to H ${}_{2}$ O ${}_{2}$ and CH ${}_{3}$ COOH (Figure 2**D**). Similarly, introduction of *YPEL2* into *moh1* Δ cells restored stress phenotypes, consistent with functional conservation between Moh1p and YPEL2 (Supplementary Information Fig. S2). Furthermore, spot assays with YPRG, glycerol, low-glucose conditions at different temperature and different media pH revealed no significant growth differences between the strains (Supplementary Information Fig. S3), indicating that *MOH1* deletion does not broadly impair metabolic flexibility or pH tolerance.

**Figure 3:**
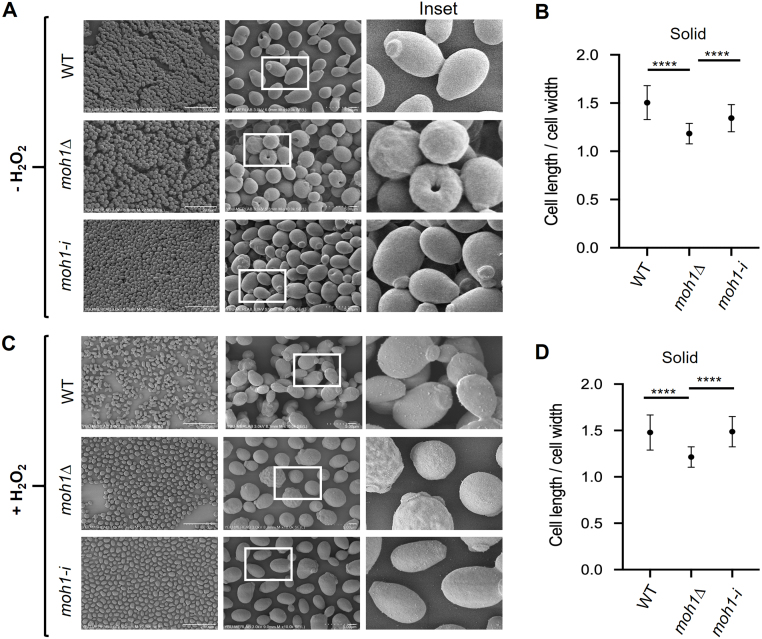
SEM of yeast strains grown on solid media in the absence or presence of H ${}_{2}$ O ${}_{2}$ as a stressor. **(A and C)** WT, *moh1* Δ , and *moh1-i* cells were grown on YPD-Agar plates containing none (-) or 3.25 mM H ${}_{2}$ O ${}_{2}$ for 40 hours at 30 ${}^{\circ }$ C. Colonies were removed and transferred into vials containing 4% glutaraldehyde in water for fixation, followed by dehydration with ethanol and air drying. Samples were coated with gold and imaged using a Scanning Electron Microscope (SEM). White squares indicate the Inset. Scale bars are shown. **(B and D)**. The cell length and width ratio of yeast strains in the absence **(B)** or the presence **(D)** of H ${}_{2}$ O ${}_{2}$ was graphed using 50 cells from images. A Student’s t-test was conducted for statistical analyses. ${}^{****}$ indicates significant difference (p < 0.0001).

Taken together, these findings suggest that the role of *MOH1* in stress survival is dependent on the type of stress encountered. While the presence of Moh1p promotes cell survival and growth under respiratory and sulfuric acid stress, the enhanced tolerance of *moh1* Δ cells to oxidative and acetic acid stress points to a potential repressive function of Moh1p under these conditions.

### Environmental stresses differentially modulate Moh1p levels

Since the expression of *MOH1* increases during re-entry into growth from the stationary phase 32, consistent with its role in the exit from the stationary phase and recovery from nutrient depletion 33, we wanted to determine whether other stressors modulate *MOH1* expression. We monitored transcript and/or protein levels under oxidative and acidic stress. We first analyzed the changes in levels of *MOH1* transcript upon short-term (45 min) (Figure 2**E**) and long-term (40 h) (Figure 2**G**) stress. RT-qPCR revealed that short-term and long-term exposure to H ${}_{2}$ O ${}_{2}$ repressed *MOH1* mRNA levels in WT and *f-moh1-i* cells (Figure 2**E** and 2**G**). On the other hand, short-term and long-term exposure to H ${}_{2}$ SO ${}_{4}$ resulted in elevated *MOH1* mRNA levels in WT and f-moh1-i cells (Supplementary Information Fig. S4A and S4D). W*e* next examined the changes in f-Moh1 protein levels with WB using the anti-Flag antibody. In Western blot (WB) analyses, for which we used Ponceau staining as the equal loading control, the Flag antibody detected a non-specific protein species that shows an electrophoretic migration similar to f-Moh1 (approximately 17 kDa). Nevertheless, consistent with the change in levels of the *MOH1* or *f-moh1* transcript, f-Moh1 protein levels were effectively decreased after both short-term and long-term H ${}_{2}$ O ${}_{2}$ treatment (Figure 2**F**–2**J**). In contrast, short-term or long-term treatment of yeast cells with H ${}_{2}$ SO ${}_{4}$ augmented f-Moh1 protein levels (Supplementary Information Fig. S4B, S4C, S4E, and S4F). Given that *moh1* Δ cells are hypersensitive to H ${}_{2}$ SO ${}_{4}$ (Figure 2**B**), this increase is consistent with a protective role for Moh1p under strong acid stress.

These observations suggest that Moh1p levels are dynamically regulated in response to stress, with distinct stress conditions exerting differential effects on its abundance. Specifically, oxidative stress suppresses *MOH1* expression and Moh1p synthesis, whereas acid stress induces them, indicating a tuning mechanism that enables cells to adjust Moh1p levels to promote survival under diverse environmental challenges.

### *MOH1* deletion alters cell shape and surface topology

*moh1* Δ cells exhibited a pronounced tendency to form clumps compared to WT cells when viewed with light microscopy (Supplementary Information Fig. S5). To further examine cellular morphology, we employed scanning electron microscopy (SEM), a technique widely used to investigate the surface ultrastructure of biological specimens 67, 68, to analyze the surface structure of WT, *moh1* Δ , and *moh1-i* strains grown on agar plates (Figure 3) and in liquid culture (Supplementary Information Fig. S6). To assess potential growth stage-dependent differences, both logarithmic-phase (Supplementary Information Fig. S6A-D) and stationary-phase (Supplementary Information Fig. S6E-H) cultures were examined. Additionally, to determine whether oxidative stress influences morphological features, the strains were also cultured in the presence of H ${}_{2}$ O ${}_{2}$ .

Cell morphology was evaluated based on the length-to-width ratio. Under all tested conditions, WT and *moh1-i* cells retained their characteristic ovoid shape, with an average ratio of ∼ 1.5. In contrast, *moh1* Δ cells appeared significantly more spherical, with ratios approaching 1 (Figure 3 and Supplementary Information Fig. S6). The morphological differences between *moh1* Δ and the control strains were even more evident in surface topography. While WT and *moh1-i* cells displayed smooth surfaces under all growth conditions or H ${}_{2}$ O ${}_{2}$ exposure, *moh1* Δ cells showed roughened surfaces with prominent protrusions and indentations (Figure 3 and Supplementary Information Fig. S6).

These findings demonstrate that deletion of *MOH1* results in constitutive morphological abnormalities, characterized by rounder cells with irregular surface structures and a tendency to clump, irrespective of growth phase or oxidative stress. This points to a critical role of Moh1p in maintenance of cellular architecture, while also implicating it in stress response and/or adaptive processes.

### Effects of *MOH1* deletion on the transcriptomic profile of *S. cerevisiae*

To gain insight into the molecular mechanisms that underly the effects of *MOH1* deletion, we compared the transcriptomic profiles of the WT and *moh1* Δ strains by high-throughput RNA sequencing (RNA-Seq) of cells from spot tests. Differentially expressed genes (DEGs) were identified using the DESeq2 package in R. Genes were considered differentially expressed if they exhibited a log ${}_{2}$ fold change ≤ −0.6 or ≥ +0.6 and an adjusted p-value below 0.05.

**Figure 4:**
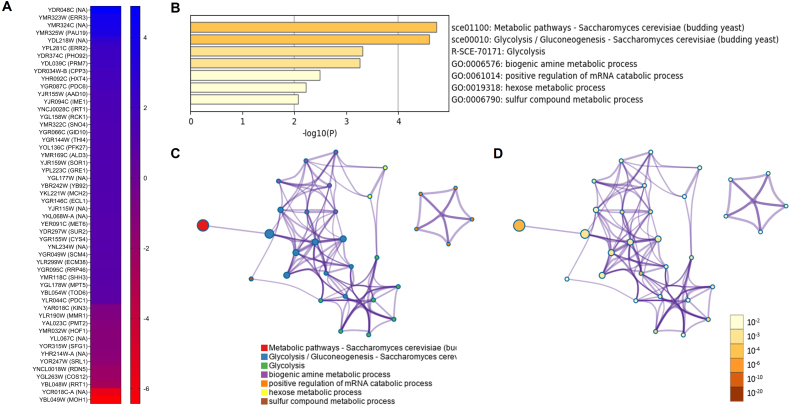
Identification of differentially expressed genes as a result of *MOH1* deletion by RNA-Seq. Total RNA of WT and *moh1* Δ cells from spot tests on YPD plates was subjected to high-throughput RNA sequencing. RNA-Seq results were analyzed using the DESeq2 package in R, and genes were considered differentially expressed if they exhibited a log ${}_{2}$ fold change ≤ −0.6 or ≥ +0.6 and an adjusted p-value below 0.05. **(A)** Heatmap of DEGs, **(B–D)** Metascape results of DEGs are presented as **(B)** Enriched terms, **(C)** Network of enriched term nodes, and **(D)** Enriched term nodes colored by p-value.

We identified 52 DEGs in the *moh1* Δ strain relative to WT, including *MOH1* as expected (Figure 4**A**, Supplementary Information Table S2). Of these, while 39 genes were upregulated, 13 genes were downregulated. According to Saccharomyces Genome Database (SGD; https://www.yeastgenome.org/), all DEGs are stress-responsive (Supplementary Information Table S2) except the long non-coding RNA *YNCJ0028C* (*IRT1*), and the 5S ribosomal RNA *YNCL0018W* (*RDN5-1*). Using RT-qPCR, we verified the differential expression of *YMR169C* (*ALD3*), *YPL223C* (*GRE1*), and *YOR247W* (*SRL1*) (Supplementary Information Fig. S7). These genes were selected due to their association with stress responses and cell wall integrity 34, 69–71.

To explore the functional implications of these DEGs, we analyzed gene ontology (GO) terms for biological functions, using SGD, Metascape (72, https://metascape.org/), and STRING (73, https://string-db.org/) databases (Figure 4, Supplementary Information Table S3 and Fig. S8). Five DEGs corresponded to dubious ORFs (*YDR048C, YMR324C, YGL177W*, and *YHR214W-A, YCR018C-A*), while four DEGs (*YKL068W-A, YBL048W,* and *YJR115W)* encode proteins of unknown functions. The Metascape analysis of the remaining DEGs indicates enrichments in the GO-terms of metabolic pathways, glycolysis, as well as biogenic amine, hexose, sulfur compound metabolic, and mRNA catabolic processes (Figure 4**B–C**). Together with SGD Database and STRING Database results, these findings suggest that *MOH1* is involved in networks critical for balancing carbon, nitrogen, and sulfur metabolism, thereby supporting cellular physiology and morphology.

Because pathway-level enrichments were based on a relatively limited number of DEGs (44 genes with known or predicted functions), we performed manual annotation of individual genes. Among the upregulated DEGs, *YJR155W* (*AAD10*), *YMR169C* (*ALD3*), *YGR155W* (*CYS4*), *YLR299W* (*ECM38*), *YPL281C* (*ERR2*), *YMR323W* (*ERR3*), *YER091C* (*MET6*), *YGR087C* (*PDC6*), *YOL136C* (*PFK27*), *YAL023C* (*PMT2*), *YMR322C* (*SNO4*), *YJR159W* (*SOR1*), *YDR297W* (*SUR2*), *YGR144W* (*THI4*), and *YBR242W* (*YB92)*, for example, encode enzymes involved in key metabolic processes including nucleic acid, protein, lipid, and carbohydrate metabolism, suggesting that Moh1p plays a central role in metabolic regulation. Additionally, *YGL158W* (*RCK1*), which encodes a threonine protein kinase that reduces ROS levels and promotes oxidative stress tolerance 74, was also upregulated, potentially contributing to the enhanced oxidative stress resistance of *moh1* Δ cells.

Conversely, several genes critical for cytoskeletal organization, cytokinesis, vesicular trafficking, membrane integrity, and cell wall stability, including *YGL263W* (*COS12), YMR032W* (*HOF1*), *YLR190W* (*MMR1*), *YAL023C* (*PMT2*), and *YOR247W* (*SRL1*), were downregulated. Furthermore, *YOR315W* (*SFG1*), a transcription repressor of genes involved in cell wall degradation 75, 76, was also downregulated in *moh1* Δ cells. Deregulated expression of these genes may account for the morphological alterations observed in *moh1* Δ cells.

Collectively, these findings position Moh1p as a key regulator of metabolic adaptation and cellular architecture.

### *MOH1* deletion alters the biomolecular composition of *S. cerevisiae*

Changes in cellular morphology and expression of genes related to lipid, protein, and carbohydrate metabolism suggest that the macromolecular composition and organization in *moh1* Δ cells are altered. To assess these biochemical changes, we employed FTIR, which provides a comprehensive biochemical fingerprint of cells by capturing unique vibrational spectra of proteins, lipids, and polysaccharides 77–85. FTIR enables both quantitative and qualitative assessment of macromolecular composition and organization 77–85 and has been widely applied to monitor biochemical changes in yeast under various conditions, including stress and apoptosis 86–94.

**Figure 5:**
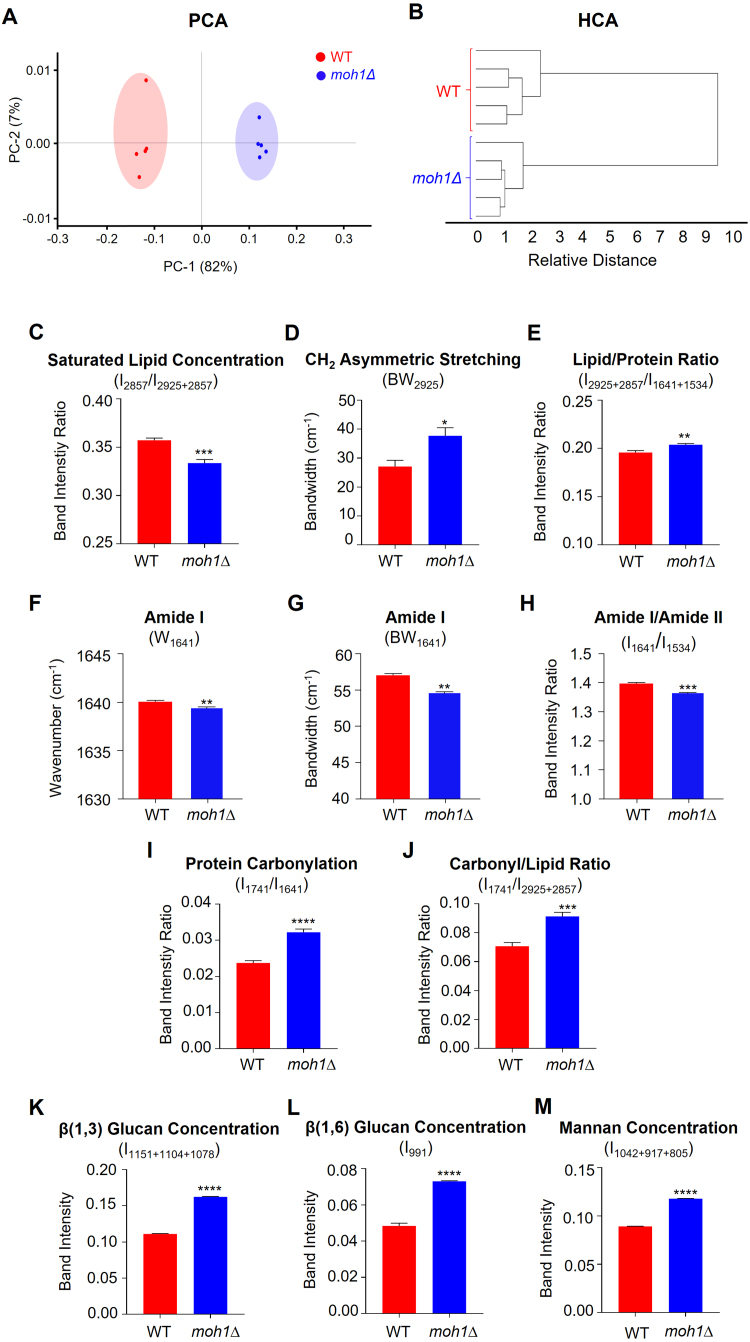
Comparative analyses of WT and *moh1* Δ cells with FTIR . **(A–M)** WT or *moh1* Δ cells spotted on the YPD-Agar plates were incubated at 30 ${}^{\circ }$ C for 40 hours. Cells were scraped from the agar plate into sterile distilled water and centrifuged at 1000 rpm for 5 minutes to form a pellet. After washing with distilled water, 2 × 10 ${}^{8}$ cells were concentrated, placed on an ATR crystal, and dried with N ${}_{2}$ for 5 min. Five biological replicates of WT and *moh1* Δ cells were subjected to FTIR analyses as two technical repeats. **(A)** PCA score, **(B)** HCA dendrogram. FTIR results of the WT and *moh1* Δ strains **(C–M)** were quantified as band intensity ratios, band width or band intensity for **(C)** saturated lipid concentrations, **(D)** CH ${}_{2}$ asymmetric stretching, **(E)** lipid/protein ratio, **(F)** amide I **(G)** amide I, **(H)** amide I/amide II, **(I)** protein carbonylation, **(J)** carbonyl/lipid ratios, **(K)** β -(1,3)-glucan concentration, **(L)** β -(1,6)-glucan concentration, and **(M)** mannan concentration. The results were presented as the mean ± S.E.M. with a student’s t-test for the statistical significance of the quantitative spectral results with ${}^{*}$ p < 0.05, ${}^{**}$ p < 0.01, ${}^{****}$ p < 0.0001.

Given the high dimensionality of IR spectral data, we applied principal component analysis (PCA) to reduce data complexity and visualize strain-specific spectral differences 95–99. The PCA results demonstrated a clear separation between WT and *moh1* Δ cells, explaining 89% of the total variance (PC1: 82%, PC2: 7%), which reflects substantial compositional divergence between the two strains (Figure 5**A**). Hierarchical clustering analysis (HCA) 100 further supported these findings by revealing distinct clustering patterns for WT and *moh1* Δ strains (Figure 5**B**). The corresponding IR band assignments for the spectral features are provided in Supplementary Information Table S4.

Quantitative comparisons of spectral bands of lipid regions indicate significant reductions in saturated fatty acids and an increase in bandwidth values of the CH ${}_{2}$ asymmetric stretching band, along with increased lipid/protein ratio in *moh1* Δ cells (Figure 5**C–E**). These changes point to altered lipid levels, modified composition and packing, and reorganization of membrane structures, processes critical for cellular stress adaptation and response 101–103. FTIR analysis also revealed a change in protein secondary structures, indicated by increases in the amide I and amide II band intensities, as well as an altered protein environment reflected in a decrease in the Amide I/Amide I + Amide II ratio (Figure 5**F–H**) 104–106. Moreover, elevated protein carbonylation and an increased carbonyl/lipid ratio indicate enhanced oxidative damage to both proteins and lipids (Figure 5**I–J**) 107, 108. Notably, *moh1* Δ cells displayed elevated levels of polysaccharides, including β -(1,3)-glucan, β -(1,6)-glucan, and mannan (Figure 5**K–M**), features that are hallmarks of a remodeled cell wall 109, 110.

Together, these findings demonstrate that *MOH1* deletion leads to widespread biochemical and structural changes, including alterations in lipid and protein profiles, oxidative damage response, membrane reorganization, and cell wall remodeling, that likely underlie the selective stress adaptations observed in *moh1* Δ cells.

### *MOH1* deletion reduces intracellular ROS by limiting H ${}_{2}$ O ${}_{2}$ uptake

Increased resistance to H ${}_{2}$ O ${}_{2}$ in *moh1* Δ cells suggests that Moh1p is directly involved in mitochondrial ROS production and/or clearance, or indirectly through cell wall remodeling, which is critical for reducing/blocking H ${}_{2}$ O ${}_{2}$ penetration/uptake into the cell. Previous studies indicated that H ${}_{2}$ O ${}_{2}$ produced intracellularly or provided exogenously activates cellular responses that enhance resistance to oxidative stress by upregulating antioxidant enzymes, including catalase 111, which catalyzes the decomposition of H ${}_{2}$ O ${}_{2}$ to water and oxygen 112. However, at elevated levels, H ${}_{2}$ O ${}_{2}$ represses catalase activity, resulting in cell death 113.

**Figure 6:**
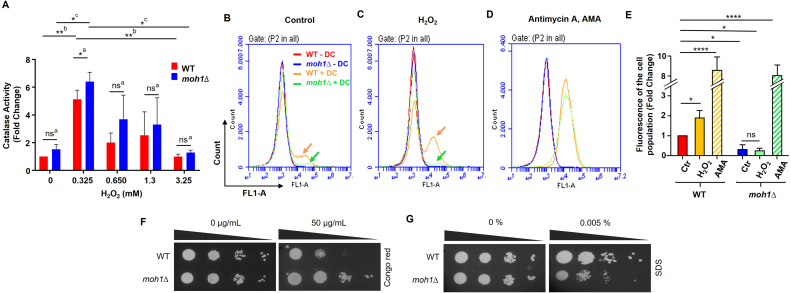
The effects of H ${}_{2}$ O ${}_{2}$ on intracellular ROS levels. **(A)** WT and *moh1* Δ strains grown in YPD with an OD ${}_{600}$ of 0.4–0.6 were treated without (0), or with 0.325, 0.650, 1.3, or 3.25 mM H ${}_{2}$ O ${}_{2}$ . Cells were then processed for and subjected to a catalase activity assay. The graph presented as mean ± S.E.M. of three independent determinations indicates fold changes in catalase activity of WT cells in the absence of H ${}_{2}$ O ${}_{2}$ (0), which was set to one. Statistical significance of the results was determined using Student’s t-test (*p < 0.05, and **p < 0.01); ns denotes non-significant. ${}^{a}$ indicates significant change compared to WT at indicated concentrations of H ${}_{2}$ O ${}_{2}$ ; ${}^{b}$ denotes responses observed with WT cells changes at 0.325 and 3.25 mM H ${}_{2}$ O ${}_{2}$ ; and ${}^{c}$ indicates significant changes responses observed with the moh1 Δ strain at 0.325 and 3.25 mM H ${}_{2}$ O ${}_{2}$ . **(B–E)** WT and *moh1* Δ cells grown in YPD with an OD ${}_{600}$ of 0.4–0.6 were treated without (control), or with 3.25 mM H ${}_{2}$ O ${}_{2}$ , or 25 μ g/mL Antimycin A (AMA) for 45 minutes. Cells were treated without or with 20 μ M reactive oxygen species indicator H ${}_{2}$ DCFDA (DC) for 45 minutes at 30 ${}^{\circ }$ C. Cells were then washed twice with PBS by centrifugation at 6000 × g for 3 minutes. **(B-D)** Fluorescence intensity was measured with a flow cytometer by using the FL1-A channel with excitation at 488 nm and emission at 525 nm. A minimum of 100,000 events was recorded per sample. Representative images from the same experiment, conducted as three biological replicates, are shown. **(E)** The graph indicates fold changes in fluorescence of the cell population normalized to the WT control, which was set to one. Results are presented as mean ± S.E.M. of three independent determinations, and statistical significance of the quantitative spectral results was determined using Student’s t-test ( ${}^{*}$ p < 0.05, ${}^{**}$ p < 0.01, and ${}^{****}$ p < 0.001); ns denotes non-significant. **(F)** WT and *moh1* Δ cells spotted on YPD-Agar plates containing 0 or 50 μ g/mL Congo red, or **(G)** 0% or 0.005% SDS with 10-fold serial dilutions (Black triangles). Cells were incubated at 30 ${}^{\circ }$ C for 40 hours and photographed.

Indeed, H ${}_{2}$ O ${}_{2}$ treatment under short-term stress (45 min) at low concentration (0.325 mM) activated catalase activity in WT and *moh1* Δ strains but represses it at high concentration (3.25 mM), as we have used throughout this study, to those levels observed without treatment (Figure 6**A**). Under the high H ${}_{2}$ O ${}_{2}$ concentration used here, the suppression of catalase activity, thereby minimization of differences in detoxification, in both strains provided an opportunity to assess whether the remodeled cell wall reduces/blocks H ${}_{2}$ O ${}_{2}$ uptake/penetration into the cell. To examine this issue, we monitored relative ROS levels in WT and *moh1* Δ cells, with or without short-term H ${}_{2}$ O ${}_{2}$ treatment (Figure 6**B–C** and 6**E**). As a reporter for ROS levels, we used 2’-7’-Dichlorodihydrofluorescein diacetate (H ${}_{2}$ DCFDA), which, upon oxidation, is converted into a fluorescent dye (2’,7’-dichlorofluorescein) and can be detected quantitatively by flow cytometry 114. To specifically assess mitochondrial ROS contribution, we included antimycin A (AMA), a well-established inducer of mitochondrial ROS by inhibiting complex III of the electron transport system 115, as a positive control. Under AMA treatment, both WT and *moh1* Δ cells exhibited comparable ROS levels, indicating that mitochondrial ROS generation is not significantly altered in the absence of *MOH1* (Figure 6**D**). On the other hand, in the absence of H ${}_{2}$ O ${}_{2}$ , *moh1* Δ cells exhibited lower basal ROS levels compared to WT cells (Figure 6**B** and 6**E**), suggesting a role for Moh1p in basal ROS generation. Upon exposure to H ${}_{2}$ O ${}_{2}$ , ROS levels increased substantially in WT cells but remained largely unchanged in *moh1* Δ cells (Figure 6**C** and 6**E**). In contrast, treatment with AMA induced comparable increases in ROS levels in both strains (Figure 6**D**). These results indicate that the reduced ROS accumulation observed in *moh1* Δ cells under H ${}_{2}$ O ${}_{2}$ treatment is not due to impaired mitochondrial ROS production but rather reflect reduced cellular uptake of H ${}_{2}$ O ${}_{2}$ . Together with FTIR and SEM data, these findings suggest that the remodeled cell wall of *moh1* Δ cells may limit H ${}_{2}$ O ${}_{2}$ permeability/uptake, thereby conferring resistance to oxidative stress induced with H ${}_{2}$ O ${}_{2}$ .

To further assess whether altered cell envelope properties contribute to the reduced intracellular ROS accumulation observed in *moh1* Δ cells, we examined their sensitivity to cell wall- and membrane-perturbing agents. In spot assays (Figure 6**F** and 6**G**; Supplementary Information, Fig. S11), *moh1* Δ cells exhibited increased resistance to Congo red, a dye that binds β -glucan and interferes with cell wall assembly 116–118, but increased sensitivity to Sodium Dodecyl Sulfate (SDS), a detergent that disrupts plasma membrane integrity 119, 120. The resistance to Congo red, despite increased β -glucan levels as indicated by FTIR, suggests reduced accessibility of cell wall polysaccharides, likely due to altered wall organization and mannan masking a results of increased mannan concentrations 116, 117 as also observed with our FTIR analysis. In contrast, the heightened sensitivity to SDS indicates compromised membrane integrity or defective coordination between the cell wall and plasma membrane. These findings are consistent with our FTIR and SEM results and suggest that *MOH1* deletion leads to cell envelope remodeling that likely alters cell permeability to H ${}_{2}$ O ${}_{2}$ .

## DISCUSSION

We present evidence that deletion of *MOH1* triggers coordinated molecular changes that reshape the physiology and cellular architecture of *S. cerevisiae*. Phenotypic assays reveal stress-specific survival responses in the *moh1* Δ strain. Integrated RNA-Seq and FTIR analyses demonstrate that deletion of *MOH1* results in transcriptional reprogramming and metabolic remodeling, reflected in altered lipid, protein, and cell wall polysaccharide composition and are consistent morphological changes observed by SEM independently of stress. Functional assays further show that increased resistance of *moh1* Δ cells to H ${}_{2}$ O ${}_{2}$ is not due to enhanced ROS detoxification but rather to reduced intracellular ROS accumulation. Together with Congo red resistance and SDS sensitivity, these results indicate altered cell envelope organization and decreased permeability to exogenous H ${}_{2}$ O ${}_{2}$ . We therefore propose that Moh1p links gene expression and metabolism to cell envelope structure, thereby modulating cell envelope permeability and conferring selective stress resistance.

Our findings identify *MOH1* as a key regulator of cell envelope homeostasis, linking metabolic state, gene expression, and structural organization to stress adaptation in *S. cerevisiae*. RNA-Seq analysis revealed coordinated transcriptional changes in *moh1* Δ cells affecting metabolism, redox balance, and cellular architecture. Although relatively few genes were differentially expressed, their functional clustering provides important mechanistic insight. Upregulation of glycolytic and sugar metabolism genes, including *PFK27, PDC6, SOR1, ERR2, and ERR3*, suggests metabolic reprogramming to sustain energy production and biosynthetic demands associated with cell envelope remodeling 121. Consistent with this, our FTIR analyses indicated alterations in lipid and carbohydrate composition, supporting a role for Moh1p in maintaining the metabolic balance required for proper envelope organization. This is accompanied by enrichment of sulfur metabolism genes including *MET6* and *CYS4*, likely reflecting increased glutathione-associated redox buffering capacity and compensatory maintenance of cellular homeostasis. In addition, upregulation of oxidative stress-related *ALD3*, *GRE1*, and *RCK1* expressions, is consistent with activation of environmental stress response pathways 121–123. While these transcriptional changes may contribute to improved oxidative stress tolerance, they are not sufficient to explain the observed H ${}_{2}$ O ${}_{2}$ resistance. Instead, functional assays demonstrate that reduced intracellular ROS accumulation in *moh1* Δ cells is primarily due to decreased uptake of exogenous H ${}_{2}$ O ${}_{2}$ rather than altered mitochondrial ROS production. This is supported by comparable ROS responses in WT and *moh1* Δ cells following antimycin A treatment, indicating that mitochondrial ROS generation remains intact. Under the high H ${}_{2}$ O ${}_{2}$ conditions used in this study, catalase activity is suppressed in both strains, further supporting permeability rather than detoxification as critical contributor of intracellular ROS levels.

Consistent with this, *moh1* Δ cells display resistance to Congo red but sensitivity to SDS. Resistance to Congo red suggests reduced accessibility of β -glucan due to increased mannan masking and altered wall organization, while SDS sensitivity indicates compromised plasma membrane integrity or defective coordination between the membrane and cell wall. Together, these findings suggest that *MOH1* is required for proper cell envelope organization and permeability control. Indeed, transcriptional downregulation of *HOF1*, *SRL1*, *COS12*, and *PMT2* involved in cytokinesis, vesicular trafficking, and cell wall integrity, and of SFG1 further support deregulated cell wall remodeling. *moh1* Δ cells exhibit stress-specific phenotypes, including increased resistance to CH ${}_{3}$ COOH and heightened sensitivity to NaN ${}_{3}$ and H ${}_{2}$ SO ${}_{4}$ . CH ${}_{3}$ COOH induces cytoplasmic acidification and oxidative stress, whereas NaN ${}_{3}$ inhibits mitochondrial respiration and H ${}_{2}$ SO ${}_{4}$ causes strong acid and membrane stress 124, 125. These differential responses suggest reprogramming of stress adaptation pathways, resulting in functionally altered envelope remodeling and selective resistance to stressors. Future studies combining Moh1p with membrane structural mutants will be important for further elucidating the mechanistic role of Moh1p in cellular envelope regulation and responses to environmental stress.

Although it is evident that Moh1p plays a role in the physiology and structural organization of *S. cerevisiae*, the mechanisms by which it functions remain unclear. Our *in silico* analyses indicate that Moh1p, like other members of the YPEL protein family, contains a single structural domain: the Yippee domain (Figure 1). Homology modeling, aimed at predicting the Moh1p function through the Yippee domain, reveals structural similarities with Yippee-like domains, MeDIY 54 or the CULT/ β -tent fold 41, of proteins from both prokaryotes and eukaryotes, and are associated with oxidoreductase, RNA binding/hydrolysis, and chaperone activities, suggesting an evolutionary link 1, 2, 41, 54. One of the key challenges in understanding the biological function of Yippee domains is the identification of endogenous ligand(s) 41. While the Yippee domain is structurally conserved, differences in amino acid composition across various proteins may result in distinct protein interaction profiles and specialized binding surfaces, leading to selective ligand recognition. MSRA and MSRB oxidoreductase enzymes, for example, reduce methionine sulfoxide to generate unmodified methionine, thereby contributing to antioxidant defense 58, 126, 127. Despite the Yippee domain, it is unlikely that Moh1p, like other YPEL proteins 30, is a methionine-sulfoxide reductase, as it lacks the invariably conserved cysteine-containing motifs essential for the catalytic activity of MSRA (GCPWG) or the MSRB (RXCXN) 58. On the other hand, the MeDIY domain of Mis18 proteins is shown to be a critical interacting surface for dimerization/oligomerization of the proteins, as well as CENP-A loading at the centromere for chromosome segregation 54, 128. It is therefore possible that the interaction of Moh1p with various proteins, dependent upon the metabolic state of the cell, triggers sets of events critical for the physiology and structural organization of *S. cerevisiae*.

Proteins function within dynamic networks of interacting partners whose composition changes in response to cellular and environmental cues 129, 130. Although we were unable to acertain the intracellular localization of Moh1p, likely due to misfolding of fluorescently tagged constructs, Moh1p was reported to interact with some members of primarily cytoplasmically localized molecular chaperone CCT/TRiC (Chaperonin Containing TCP-1/T-complex protein Ring Complex) and GID (Glucose-Induced Degradation complex) complexes using immuno-affinity purification coupled with mass spectrometry 131. The CCT/TRiC complex is a cytosolic type II chaperonin composed of two stacked rings, each containing eight distinct subunits (CCT1-CCT8), which mediate ATP-dependent folding of newly synthesized and stress-denatured proteins 132, 133. Through conformational changes driven by ATP binding and hydrolysis, CCT facilitates folding of key substrates involved in cytoskeletal organization, signaling, and cell cycle regulation, including actin and tubulin 132–134. Although, to our knowledge, no functional studies have established Moh1p as a regulator of these complexes, interaction of Moh1p with with CCT2 and CCT3 subunit proteins 131 suggests a potential role in modulating chaperone-mediated protein folding.

The conserved GID and GID (Glucose-Induced Degradation complex) complex is a multi-subunit E3 ubiquitin ligase composed of core Gid1, Gid2, Gid5, Gid7, Gid8, and Gid9 135 along with Gid4 135, Gid10 136, and Gid11 137, 138 subunits that function in a condition-specific manner. The GID complex regulates proteasomal degradation of metabolic enzymes, particularly during transitions between gluconeogenesis and glycolysis 135–139. Upon glucose reintroduction, activation of the complex through the incorporation of substrate receptors like Gid4 enables the ubiquitination and degradation of enzymes such as Fbp1, Pck1, Mdh2, and Icl1 140, 141. Moh1p was reported to interact with the multiple core GID subunits by immuno-affinity purification coupled with mass spectrometry 131. We observed *GID10* expression is elevated in the *moh1* Δ strain (Figure 4**A** and Supplementary Information Table S2). Gid10, a stress-induced substrate receptor, shares substrate specificity with Gid4 128, but also targets distinct proteins such as Art2, involved in plasma membrane quality control 142, 143. These findings imply an altered GID-dependent proteolysis in *moh1* Δ cells under stress, linking GID activity to cellular envelope regulation 142, 143.

The interaction of Moh1p with the CCT/TRiC and GID complexes suggests a regulatory role in coordinating selective protein folding and degradation during environmental adaptation. This aligns with RNA-Seq data showing a metabolic shift toward glycolysis in *moh1* Δ cells, accompanied by changes in lipid, protein, and polysaccharide composition and corresponding cell wall alterations observed by SEM. These findings suggest that *moh1* Δ cells may reconfigure stress defenses through altered CCT and GID activity. The involvement of Moh1p in cellular response to various stressors could entail distinct integrated mechanisms ranging from transcription to post-translational processes, with proteostasis at their core. While detailed mechanistic analyses of each stress response would require targeted biochemical and genetic approaches, Moh1p may function as an adaptor linking CCT-mediated folding and GID-mediated degradation, thereby influencing cell fate under stress. Stress-dependent changes in Moh1p levels, as we observed in response to H ${}_{2}$ O ${}_{2}$ , or increases induced by H ${}_{2}$ SO ${}_{4}$ , could modulate its interactions with these complexes, leading to a functional state in the complexes permissive or inhibitory for selective protein folding and degradation. Additionally, Moh1p may act as a sensor for monitoring pH changes or ROS levels that potentially trigger conformational alterations, influencing association with or dissociation from the CCT/GID complexes. Moh1p was also reported to be phosphorylated at two serine residues of the immediate amino-terminus region of the protein 144. Such modifications could alter proteostasis and drive metabolic reprogramming by sterically or electrostatically hindering the interaction of Moh1p with the CCT/GID complexes. Further studies aimed at identifying ligands, direct binding partners, and spatial-temporal dynamics of Moh1p across stress gradients will be crucial in elucidating its mechanistic role within the proteostasis network of *S. cerevisiae*.

In summary, our results indicate that deletion of *MOH1* causes coordinated transcriptional, biochemical, and structural changes, including pronounced alterations in cell envelope properties and permeability to stressors. Together, these findings identify Moh1p as a critical link between intracellular metabolic processes and the cell surface, enabling selective resistance to environmental stress.

## MATERIAL AND METHODS

### Homology modeling

The alignment of amino acid sequences was carried out using Jalview 43 program (https://www.jalview.org/) with the ClustalOmega 44 plug-in (https://www.ebi.ac.uk/Tools/msa/clustalo/). To predict the similarities between the secondary structures of Moh1p and YPEL2, we used the jPred4 45 server ( http://www.compbio.dundee.ac.uk/jpred4/index.htmL). For the tertiary structure prediction and superimposition of the tertiary structures of Moh1p and YPEL2, we used the AlphaFold 46, 47 server (https://alphafold.ebi.ac.uk/) with the ChimeraX molecular visualization 48, 49 program (https://www.cgl.ucsf.edu/chimerax/). For the homology modeling f YPEL2 and Moh1 proteins, we used Phyre2 50 web tool (http://www.sbg.bio.ic.ac.uk/~phyre2/htmL/page.cgi?id=index).

### Yeast strains and growth conditions

Strains that are used in this study are given in Table 1. *S.cerevisiae* BY4741 (*MATa*, *his3* Δ *1, leu2* Δ *0, met15* Δ *0, ura3* Δ *0*) strain as the wild-type strain (WT) and BY4741 *MOH1* knockout mutant strain (*moh1* Δ ; *MATa, his3* Δ *1, leu2* Δ *0, met15* Δ *0, ura3* Δ *0, Moh1* Δ *::KanMX4*) were purchased from Dharmacon (HorizonDiscovery Inc., UK). For the growth of cell strains, we used a yeast extract-peptone-dextrose (YPD) medium containing 1% yeast extract (Sigma, Germany, 70161), 2% dextrose (Sigma, Germany, 49159), and 2% peptone (Sigma, Germany, 912489). For the colony selection after transformation, we used a synthetic complex medium without uracil (SC-URA) that contains 0.67% yeast nitrogen base without amino acids (Sigma, Germany, Y0626), 2% dextrose, and 0.192% yeast synthetic drop-out medium supplements without uracil and YPD with 5-FOA (1 mg/mL 5-FOA; F9001, Zymo Research, USA).

To induce stress, we used two conditions: short-term and long-term. In the short-term stress condition, cells were grown overnight in YPD at 30 ${}^{\circ }$ C with shaking at 180 rpm. Cells in log phase were then subcultured into fresh YPD at a 1:100 dilution and grown at 30 ${}^{\circ }$ C until the OD ${}_{600}$ reached 0.4–0.6. A stress inducer was added to the medium, and cells were incubated for 45 minutes. Cells were harvested by centrifugation, pelleted, and processed for subsequent experiments. For the long-term stress condition, the autoclaved YPD-Agar medium was cooled down to 55 ${}^{\circ }$ C, and a stress inducer was added to stress agar plates. For spot tests, cells grown overnight in YPD at 30 ${}^{\circ }$ C with shaking at 180 rpm were sub-cultured into fresh YPD at a 1:100 dilution and grown until OD ${}_{600}$ reached 0.4. Cells were subsequently spotted at serial dilution on agar plates containing a stress inducer, and the plates were incubated at 30 ${}^{\circ }$ C for 40 hours. Cells were then collected with a scraper into sterile water and pelleted for subsequent experimental procedures.

**Table 1:** Cell strains used in this study .

**Name**	**Genotype**	**Abbrevation**	**Source**
BY4741	MATa *his3* Δ *1 leu2* Δ *0 met15* Δ *0 ura3* Δ *0*	WT	Dharmacon
*moh1* Δ *-*BY4741	BY474 *moh1* Δ *::KanMX4*	*moh1* Δ	Dharmacon
*moh1* Δ *-*BY4741*+URA3*	BY4741 *moh1* Δ *::URA3*	*ura3-i*	This study
*moh1* Δ *-*BY4741*+WT-MOH1*	BY4741 *moh1* Δ *::MOH1*	*moh1-i*	This study
*moh1* Δ *-*BY4741*+flag-MOH1*	BY4741 moh1 Δ ::flag-MOH1	*f-moh1-i*	This study

As stress inducers, we used sulfuric acid (Merck, Germany, 1.00713), hydrogen peroxide (Merck, Germany, 107209), acetic acid (Sigma, Germany, A6283), and sodium azide (Sigma, Germany, S2002) by adding them to YPD medium.

### Establishment of yeast strains

#### Cloning into the upstream and downstream homology arms of the pBS-KS(-) vector

The *moh1* Δ yeast strain contains the KanMX4 selector module in the *MOH1* locus. For the generation of the *moh-i* or *f-moh-i* strain, we initially replaced the KanR gene in the KanMX selector module with *URA3* (orotidine-5 ${}^{\prime }$ -phosphate decarboxylase) as the selector module in the *moh1* Δ strain. To accomplish this, we initially cloned the upstream (UPS) and downstream (DNS) homology arms (about 200 bp) of the KanR gene generated by PCR using the *moh1* Δ genomic DNA as the template and specific primer sets (Supplementary Information Table S5) with restriction enzyme sites into the pBS-KS(-) vector to generate pBSKS-UPS-DNS. We subsequently inserted the URA3 gene generated with PCR by using the pRS314-URA3 vector (a gift from Francisco Malagon; Addgene plasmid #11004; http://n2t.net/addgene:11004; RRID: Addgene_11004) DNA as the template and URA3-specific primers with restriction enzyme sites into pBSKS-UPS-DNS to obtain the pBSKS-UPS-URA3-DNS vector.

#### Insertion into the yeast genome

For the insertion of the UPS-URA3-DNS selector module into the *moh1* Δ genome, the UPS-URA3-DNS DNA fragment generated with PCR using pBSKS-UPS-URA3-DNS as the template was transformed into the *moh1* Δ strain for homologous recombination. For transformation, a single colony of the *moh1* Δ strain was grown in YPD overnight at 30 ${}^{\circ }$ C with shaking at 180 rpm. The overnight culture was then subcultured into fresh YPD at a 1:100 dilution and grown until OD ${}_{600}$ reached 0.4–0.6. Cells were pelleted at 4000 rpm for 5 minutes and washed with sterile distilled water. After centrifugation, cells were resuspended in 0.1 M LiAc solution and centrifuged at 12000 rpm for 10 seconds. The cell pellet was resuspended in 0.1 LiAc solution and placed on ice. Single-stranded salmon sperm DNA (ssDNA) was boiled at 98 ${}^{\circ }$ C for 10 minutes and incubated on ice for 10 minutes. The transformation was carried out by sequential addition of 50% (w/v) PEG (Sigma, Germany, 202444), 1 M LiAc, 2 mg/mL ssDNA, the PCR product, and the cell suspension in a fresh tube and vortexing it for 2 minutes. The suspension was then plated onto SC-URA plates, and cells were grown at 30 ${}^{\circ }$ C for 3 days for colony formation.

#### Screening of transformants

Single colonies were placed into SC-URA selective medium to screen transformants. Overnight-grown cells were centrifuged, and the cell pellet was resuspended in yeast lysis buffer containing 10 mM Tris (pH = 8), 0.2% Triton X-100, 1% SDS, 100 mM NaCl, and 1 mM EDTA. Phenol:chloroform:isoamyl alcohol (25:24:1, basic) and acid-washed glass beads (Sigma, Germany, G9268) were added to resuspended cells. The mixture was vortexed for 4 minutes. Tris-EDTA (TE) buffer was added to the mixture and was centrifuged at 6500 rpm for 2 minutes. The aqueous phase was transferred into a new centrifuge tube and washed with 100% ethanol. DNA was pelleted by centrifugation at 12000 rpm for 2 minutes. The pellet was dissolved in TE buffer, precipitated with ethanol and 3 M ammonium acetate. The genomic DNA pellet was air-dried for 15 minutes and resuspended in water. The genomic DNA was used for PCR with primer sets specific to (1) KanR, (2) URA3, (3) URA3 and upstream sequences of the UPS homology arm in the genome, (4) sequences at the upstream and downstream of UPS and DNS homology arms, respectively, in the genome (Supplementary Information Fig. S9A, S9B and Table S5). After PCR confirmation, samples were sequenced to verify PCR results.

#### Insertion of the WT-MOH1 or Flag-MOH1 gene into the yeast genome

To generate yeast strains bearing WT-*MOH1* (*moh1-i*) or *Flag*-*MOH1* (f-moh1-i), we used the same procedure to generate *moh1* Δ with *URA3* as described above, such that genes with upstream and downstream homology arms of the *MOH1* locus were amplified with PCR. Amplicons were then transformed into the *ura3-i* strain as described above. Cell suspensions were then plated onto YPD. After one day of growth, plates were replica-plated onto YPD containing 5-fluoroorotic acid 145 and placed in a 30 ${}^{\circ }$ C incubator for three days for colony formation. Following PCR screening of transformants with primers specific to the gene of interest and homology arms as described above (Supplementary Information Fig. S9C, S9D and Table S5), positive transformants were subjected to sequencing.

### Growth in water

A single colony from WT, *moh1* Δ , *moh1-i*, and *f-moh1-i* strains was grown in YPD overnight. 50 × 10 ${}^{6}$ cells were subcultured into fresh YPD and grown for one week. After one week of growth, cells were washed twice with 1 M Sorbitol (Sigma, Germany, S1876) and once with sterile distilled water. Cells were then resuspended with 20 mL of sterile distilled water and incubated at 30 ${}^{\circ }$ C and 180 rpm for up to 18 days. Every second day, aliquots from these water cultures were collected for a spot test, for which 10-fold serial dilutions from each aliquot were spotted on the YPD-Agar plate. Cells were incubated at 30 ${}^{\circ }$ C for 40 hours and photographed with the ChemiDoc ${}^{\mathrm{TM}}$ MP System (BioRad, USA).

### Growth under stress conditions

A single colony from WT, *moh1* Δ , *moh1-i*, and *f-moh1-i* strains grown in YPD overnight was subcultured into 20 mL of YPD at a ratio of 1:100. Cells were grown until OD ${}_{600}$ reached 0.4–0.6. Cells were counted, and 2.5 × 10 ${}^{6}$ cells/mL was used for 10-fold serial dilutions for spotting on stress agar plates as described above. Similarly cells were spotted on stress agar plates containing none, or 25–100 μ g/mL Congo red (MedChemExpress, USA, HY-D0236) or 0.005–0.01% SDS (Sodium Dodecyl Sulfate, Sigma-Aldrich, Germany, A, L3771). Plates were incubated at 30 ${}^{\circ }$ C for 40 hours. The spots on the plates were photographed with the ChemiDoc ${}^{\mathrm{TM}}$ MP system (Bio-Rad, USA).

To assess the effects of nutritional adjustments under different temperature or pH on the growth of WT and *moh1* Δ cells, 2.5 × 10 ${}^{6}$ cells/mL, with 10-fold serial dilutions (black triangles), were spotted onto the indicated agar (2%) plates: YPD (1% Yeast extract, 2% Peptone, 2% Dextrose), YPRG (1% Yeast extract, 2% Peptone, 3% Raffinose, and 2% Galactose), Glycerol (1% Yeast extract, 2% Peptone, 3% Glycerol), or low glucose (1% Yeast extract, 2% Peptone, 0.5% or 1% Glucose). Plates were incubated at 23, 30, 33, 35, and 37 ${}^{\circ }$ C for 40 hours and photographed.

For spot tests at different pH, buffered YPD agar plates prepared YPD-agar base (1% yeast extract, 2% peptone, 2% dextrose and 2% agar), sterilized by autoclaving. The molten medium was then supplemented with sterile buffer to the indicated pH while maintaining a constant final buffer strength ( ∼ 50 mM) across conditions. For pH 6–8, a citrate-phosphate system was used by combining citric acid with Na ${}_{2}$ HPO ${}_{4}$ at pH-specific ratios to achieve the target pH. Plates were incubated at 30 ${}^{\circ }$ C for 40 hours and photographed.

### Western Blot (WB)

Cells subjected to either long-term or short-term stress were collected and counted. 500 × 10 ${}^{6}$ cells were pelleted in centrifuge tubes. After washing the pellets with sterile distilled water, the cells were resuspended in 75 μ L of urea-lysis buffer (40 mM Tris, pH 6.8; 0.1 mM EDTA; 5% SDS; 9 M urea; 0.02 mg/mL bromophenol blue) and mixed with 75 μ L of glass beads. The mixture was vortexed five times for 1 minute each, with 1-minute ice breaks between vortexes. Using a heated syringe needle, the bottom of the tube was punctured and the contents transferred to a clean tube, then centrifuged at 4000 rpm for 2 minutes to remove glass beads. The mixture was centrifuged at 14,000 rpm for 15 minutes, and the supernatant was transferred to a clean tube. Equal volumes (25 μ L) of supernatant from each sample were loaded onto a 10% SDS-PAGE gel for WB analysis. After transferring proteins to a PVDF membrane (Advansta, WesternBrightTM PVDF-CL, L-08008-001) via wet transfer, the membrane was blocked with 5% skim milk in 0.1% Tris-buffered saline-Tween (TBS-T). Proteins were detected using a Flag Antibody (1:1000 dilution; M2-Flag, Sigma-Aldrich, F-1804). Specificity of detection of flag-Moh1 with the Flag antibody was initially assessed in COS7 cells, an African green monkey kidney fibroblast-like cell line as we described previously 30, which were transiently transfected with the expression vector bearing none (Vector control), MOH1 or Flag-MOH1 cDNA for 24 h (Supplementary Infomation, Fig. S12). Following three 5-minute washes with 0.1% TBS-T, the membrane was incubated with a goat anti-mouse HRP-conjugated secondary antibody (1:4000 dilution in 5% skim milk in 0.1% TBS-T, Santa Cruz Biotechnology, USA) for 1 hour at room temperature. The membrane was then treated with WesternBright ECL substrate (Advansta, K-12045-D50) in a 1:1 ratio of luminol-enhancer reagent to peroxide reagent in the dark for 2 minutes to detect proteins. The ChemiDocTM MP system (Bio-Rad, USA) was used for visualization.

### RNA isolation and RT-qPCR

Cells subjected to long- or short-term stress were collected and pelleted in centrifuge tubes. To dissolve cell pellets, 25 μ L of 20% SDS and 400 μ L of ice-cold Acetate-EDTA (AE) buffer containing 50 mM sodium acetate and 10 mM EDTA (pH = 8) were added to the cell pellets. Subsequently, 500 μ L of acidic 25:24:1 phenol:chloroform:isoamyl alcohol (PCI) solution was added to the cell suspension. The cell suspension was incubated at 65 ${}^{\circ }$ C for 15 minutes, followed by incubation on ice for 10 minutes. For phase separation, samples were centrifuged at 14000 rpm at 4 ${}^{\circ }$ C for 15 minutes, and the liquid phase was transferred to a sterile tube containing 500 μ L of PCI. The mixture was vortexed for 20 seconds and incubated on ice for 10 minutes. After centrifugation of the samples at 14,000 rpm for 15 minutes at 4 ${}^{\circ }$ C, the supernatant was transferred to a new sterile tube, followed by the addition of one-tenth volume of sodium acetate (pH 5.3) and three volumes of 100% ethanol. Samples were incubated at −80 ${}^{\circ }$ C for 30 minutes. RNA was precipitated by centrifugation at 14000 rpm at 4 ${}^{\circ }$ C for 15 minutes and dissolved in diethylpyrocarbonate (DEPC)-treated water. RNA samples were also treated with DNase I to degrade any remaining DNA. The purity and concentration of RNA were determined using a Nanodrop 2000 spectrophotometer (Thermo-Fisher Sci., California, USA) with A260/A280 and A260/A230 ratios. Isolated RNAs were used for cDNA synthesis (The RevertAid First Strand cDNA Synthesis Kit, Thermo-Fisher) with oligo (dT) ${}_{18}$ primers according to the manufacturer’s instructions. qPCR experiments were carried out with SsoAdvanced Universal SYBR Green SuperMix (Bio-Rad, USA) and transcript variant-specific primers. *YPR062W* (*FCY1*) and *YNL219C* (*ALG9*) were used as reference genes for normalization in the analyses, and the differential expression was shown as fold change using the 2 ${}^{-\Delta \Delta C_{T}}$ approach 146. RT-qPCR experiments were performed with the MIQE Guidelines 147.

### Scanning Electron Microscopy (SEM)

Samples were prepared and analyzed using SEM at the Prof. Dr. Zekiye Suludere Electron Microscope Center, Gazi University, Ankara, Türkiye. For SEM analysis, WT, *moh 1* Δ , and *moh 1-i* cells were cultured under logarithmic phase, stationary phase, and solid-growth conditions. A single colony was initially inoculated into YPD medium and incubated overnight at 30 ${}^{\circ }$ C with reciprocal shaking at 180 rpm. To obtain cells in the logarithmic phase, cultures were diluted 1:100 and grown at 30 ${}^{\circ }$ C with reciprocal shaking at 180 rpm until reaching an OD ${}_{600}$ of 0.4–0.6. At this point, half of the culture was subjected to short-term H ${}_{2}$ O ${}_{2}$ stress for 45 min while the other half served as the control. For the stationary phase, after subculturing, cells were grown for 48 hours at 30 ${}^{\circ }$ C with reciprocal shaking at 180 rpm. This was followed by short-term H ${}_{2}$ O ${}_{2}$ stress for 45 minutes, while the other half served as the control. For cells in the logarithmic and stationary phases, cultures were collected, washed twice with sterile water, and centrifuged at 1,000 rpm for 3 minutes. The resulting cell pellets were resuspended in 4% glutaraldehyde for fixation. To prepare solid cultures, cells from the logarithmic phase were counted using a hemocytometer, and 250 cells were plated on YPD-Agar plates with or without H ${}_{2}$ O ${}_{2}$ . Plates were incubated for 40 hours at 30 ${}^{\circ }$ C. From these plates, small sections containing yeast colonies were excised and transferred into vials containing 4% glutaraldehyde in water for fixation. After fixation, cells were rinsed with water. Fixed cells were dehydrated using an ascending series of ethanol (70%, 80%, 90%, and 100%) and then air-dried. The samples were treated with amyl acetate, followed by critical point drying using a Polaron CPD 7501 (Quorum Technologies, UK) under liquid carbon dioxide. Samples were coated with gold using a Polaron SC 502 sputter coater (Quorum Technologies, UK). Imaging was performed with a HITACHI SU 5000 Schottky Field Emission Scanning Electron Microscope (FE- SEM, HITACHI, Japan).

### RNA sequencing

#### RNA-Seq analysis

Two μ g of RNA extracted from WT and *moh1* Δ cells in spot tests, as described above, were used for sequencing. For RNA sequencing, RNA-Seq libraries were prepared with the BGISEQ-500 sequencing platform (Genoks, Ankara, Türkiye, through BGI Genomics, Shenzhen, People’s Republic of China) with a 100 bp paired-end sequencing approach and a sequencing depth of at least 30 million 148, 149. Results obtained after the sequencing were subjected to quality control. Contaminations in the read data, adapter sequences, sequences shorter than 30 bases, and low-quality readings were filtered out. The clean readings obtained after filtering were aligned to the latest *S. cerevisiae* genome (Ensemble 101) using STAR (Hierarchical Indexing for Spliced Alignment of Transcripts) software 150. Transcript and gene count matrices were obtained with featureCounts 151 after the alignment process. The RNA-Seq data generated in this study have been deposited in the ArrayExpress database (https://www.ebi.ac.uk/arrayexpress/) under accession number E-MTAB-16090. The count data were used to analyze differentially expressed genes (DEGs) using DeSeq2. For the DeSeq2, WT cells were used as the reference. Genes with an adjusted p-value < 0.05 and a log ${}_{2}$ fold change greater than ± 0.6 were considered differentially expressed. After DeSeq2 analysis, DEGs were listed for each group, and pathway analyses were performed. All analyses were conducted using the R language. We utilized the Metascape portal (72https://metascape.org/) using a P-value of 0.05 as the threshold.

#### Verification of RNA-Seq results with RT-qPCR

RNA samples reserved before RNA sequencing were used for the construction of cDNA libraries using the RevertAid First Strand cDNA Synthesis Kit (Thermo-Fisher) with oligo (dT)18 primers according to the manufacturer’s instructions. *ALD3, GRE1,* and *SRL1*, identified as differentially expressed genes and selected based on their cellular functions, were chosen to verify the RNA-Seq results. RT-qPCR experiments were carried out as described in Section 2.4 using transcript-specific primer sets (Supplementary Information Table S5).

### Fourier Transform-Infrared Spectroscopy (FTIR)

WT and *moh1* Δ strains were cultured overnight in YPD medium at 30 ${}^{\circ }$ C with shaking at 180 rpm. After overnight growth, cells were diluted 1:100 into fresh YPD medium. Cells grown to an OD ${}_{600}$ of 0.4–0.6 were spotted on YPD-agar plates and incubated at 30 ${}^{\circ }$ C for 40 hours. Cells were scraped from the agar plate, placed in sterile distilled water, and centrifuged at 1000 rpm for 5 minutes. The cells were washed once with distilled water, and 20 × 10 ${}^{7}$ were resuspended in 5 μ L of water. Five biological replicates of WT and *moh1* Δ cells were prepared for FTIR analysis.

FTIR readings were conducted at the East Anatolia High Technology Application and Research Center of Atatürk University in Erzurum, Türkiye, using the Attenuated Total Reflectance (ATR) mode of FTIR spectroscopy (Bruker Vertex 70, Ettlingen, Germany). To prepare samples, 3 μ L of concentrated yeast was placed on an ATR crystal, and cells were dried with N ${}_{2}$ gas for 5 minutes. For each group, five spectra were collected with two technical replicates. Spectra were acquired with 32 scans over the 4000–400 cm ${}^{-1}$ spectral range at a spectral resolution of 4 cm ${}^{-1}$ using OPUS 7.5 (Bruker, Ettlingen, Germany) software. After each measurement, the diamond crystal was cleaned with 70% ethanol and distilled water.

For qualitative and quantitative spectral analysis, all data manipulation and calculations to determine band intensity values, bandwidth, and band positions were performed using OPUS 5.5 software (Bruker Optics, Reinstetten, Germany). Quantitative band intensity (I) calculations were performed using concave rubber band baseline correction (iteration: 15; number of iterations: 128) on spectra over the entire spectral range for all samples. Band intensity ratios were used to estimate the relative concentrations of individual biomolecules within the system. When broad bands have bandwidths exceeding the separation between adjacent peaks, techniques such as Fourier deconvolution, second derivative analysis, and curve fitting are suitable for resolution enhancement 77. In this study, second-derivative analysis was applied to the spectral range of 1185–930 cm ${{}^{-}}^{1}$ to assess the contributions of mannan and glucan components. Spectral parameters related to lipid order and membrane fluidity were determined from the frequency and bandwidth of the CH ${}_{2}$ asymmetric band. To investigate variations in protein secondary structures, we also used the second derivative method 152, 153. This process began with a concave rubber band baseline correction (15 iterations; number of iterations: 128) across the full spectral range, followed by vector normalization within 1700–1600 cm ${{}^{-}}^{1}$ . Second derivative spectra were then obtained using the Savitzky-Golay algorithm (9 smoothing points) in OPUS 5.5 software (Bruker Optics, Reinstetten, Germany). The characteristic minima in these second derivative spectra correspond to secondary protein structures: α -helices (1654 cm ${{}^{-}}^{1}$ ), β -sheets (1639 cm ${{}^{-}}^{1}$ ), and β -turns (1689 cm ${{}^{-}}^{1}$ ) 154, 155. Changes in the intensity of these amide I bands in the second-derivative spectra reflect the relative proportions of secondary structural components of proteins.

Chemometric analyses, including principal component analysis (PCA) and hierarchical cluster analysis (HCA), were performed using the Unscrambler X software package (version 10.4, CAMO Software, Oslo, Norway) on spectra that had been baseline-corrected with concave rubber band correction (iteration: 15; iteration number: 128) and vector-normalized within the 4000–400 cm ${{}^{-}}^{1}$ spectral range. PCA was applied to the preprocessed spectra across the entire spectral region (4000–400 cm ${{}^{-}}^{1}$ ) to identify differences between WT and *moh1* Δ cells. The analysis used mean-centered data, full cross-validation, and the singular value decomposition (SVD) algorithm. Results were displayed as PCA score plots. To support the PCA findings, HCA was performed using Ward’s linkage algorithm combined with squared Euclidean distance measurements. The clustering results were visualized with a dendrogram.

A student’s t-test was performed using GraphPad Prism 8.0 statistics software (GraphPad, La Jolla, CA) for the statistical significance of the quantitative spectral data. The results are presented as the mean ± S.E.M., and values less than or equal to 0.05 were considered statistically significant for comparisons, *p ≤ 0.05; **p ≤ 0.01, ***p ≤ 0.001).

### Catalase activity assay

WT and *moh1* Δ strains, cultured overnight in YPD at 30 ${}^{\circ }$ C with shaking at 180 rpm, were diluted 1:100 into fresh YPD and grown to OD ${}_{600}$ = 0.4–0.6. Cultures were split into five aliquots; one was used as an untreated control, and the others were exposed to 0.325, 0.650, 1.3, or 3.25 mM H ${}_{2}$ O ${}_{2}$ for 45 min. Cells were harvested, washed with 100 mM potassium phosphate (pH 7.0) containing 0.1 mM PMSF. Cells were resuspended in 200 μ L of the same buffer and disrupted with an equal volume of acid-washed glass beads by four cycles of 45 seconds of vortexing and 45 seconds of cooling on ice. Lysates were clarified by centrifugation (13,000 × g, 10 min, 4 ${}^{\circ }$ C), and the supernatants were used as crude protein extracts.

From these crude extracts, 60–100 μ L (minimum of 80 μ g protein) of the sample was added to 2 mL of 20 mM H ${}_{2}$ O ${}_{2}$ in 50 mM KPi buffer (pH 7.0), and H ${}_{2}$ O ${}_{2}$ decomposition was monitored at 240 nm ( ${ɛ}_{240}$ = 43.6 M ${}^{-1}$ cm ${}^{-1}$ ) for 1 minute. For the catalase activity calculation, the following formulation was used:

$$\frac{\Delta OD_{240}/\min*{Volume}_{Total}*10^{6}}{{ɛ}_{240}*l*Protein\;mass\;\left(mg\right)}\begin{array}{c} \Delta OD_{240}/\min:\text{the rate of decrease in absorbance at}\\ \;\;\;\;\text{240 nm per minute}\\ {Volume}_{Total}:\text{total reaction volume (L)}\\ {ɛ}_{240}:\text{molar extinction coefficient of H}{}_{2}\text{O}{}_{2}\\ \;\text{(43.6 M}{}^{\mathrm{-1}}\text{cm}{}^{\mathrm{-1}}\text{)}\\ l:\text{path length (1 cm)}\\ Protein\;mass\left(mg\right):\text{total protein amount present in the assay} \end{array}$$

Catalase activity results are expressed as the fold change of the mean ± S.E.M. from three independent cultures, with comparisons considered statistically significant at p ≤ 0.05.

### Flow cytometry analysis

2 ${}^{\prime }$ , 7 ${}^{\prime }$ - Dichlorofluorescin diacetate (H ${}_{2}$ DCFDA or DCFDA) is a widely used fluorometric probe for detecting reactive oxygen species (ROS) within cells. H ${}_{2}$ DCFDA is a non-fluorescent, lipophilic, and cell membrane-permeable compound 114. It is recognized as a general ROS indicator, reacting with various species including hydroxyl and peroxyl radicals, peroxynitrite, and lipid hydroperoxides 114. Once inside the cell, H ${}_{2}$ DCFDA becomes fluorescent upon oxidation by cellular ROS, enabling the detection of intracellular ROS. For ROS detection with H ${}_{2}$ DCFDA, we used WT and *moh1* Δ cells. A single colony was inoculated into YPD medium and incubated overnight at 30 ${}^{\circ }$ C with shaking at 180 rpm. To obtain cells in the logarithmic phase, the overnight culture was diluted 1:100 in fresh YPD and further incubated under the same conditions until OD ${}_{600}$ reached 0.4–0.6. The culture was then divided into three equal parts: one received a treatment of 3.25 mM H ${}_{2}$ O ${}_{2}$ for 45 minutes to induce short-term oxidative stress; another was treated with 25 μ g/mL Antimycin A (Sigma, Germany, A 8674) for 45 minutes to stimulate mitochondrial ROS production as a positive control. The remaining part was left untreated as a control. After incubation with none, H ${}_{2}$ O ${}_{2}$ , or Antimycin A, each culture was split into two equal parts and transferred into microfuge tubes. One-half was incubated with 20 μ M H ${}_{2}$ DCFDA (MedChemExpress, USA, HY-D 0940) for 45 minutes at 30 ${}^{\circ }$ C in the dark to detect ROS accumulation. The other half was left untreated to serve as an unstained control. Following staining, cells were washed twice with PBS by centrifugation at 6000 × g for 3 minutes. Fluorescence intensity was then measured using a BD Accuri C6 Plus Flow Cytometer (BD Biosciences, USA). Since H ${}_{2}$ DCFDA fluoresces upon oxidation with excitation at 488 nm and emission at 525 nm, the FL1-A channel was used for detection. A minimum of 100,000 events was recorded per sample. A sample gating strategy is provided in Supplementary Information Fig. S10. Data acquisition and analysis were performed using BD Accuri C6 software (BD Biosciences, USA).

## AUTHORS CONTRIBUTIONS

Çağla Ece Olgun and Gizem Turan Duman have contributed equally.

Çağla Ece Olgun: conceptualization, data curation, formal analysis, investigation, funding acquisition, methodology, writing original draft, and editing.

Gizem Turan Duman: data curation, investigation, methodology, writing original draft, and editing.

Gizem Güpür: data curation, investigation, methodology, writing original draft, and editing.

Hamit İzgi: RNA-Seq Analysis

Mariam Huda: methodology

Demet Çetin: Scanning Electron Microscopy Analysis

Zekiye Suludere: Scanning Electron Microscopy Analysis

Fatma Küçük Baloğlu: FTIR analysis

Ayşe Koca Çaydaşı: methodology, formal analysis, writing original draft, and editing

Mesut Muyan: conceptualization, data curation, formal analysis, supervision, funding acquisition, methodology, writing original draft, editing, and project administration.

## SUPPLEMENTAL MATERIAL

All supplemental data for this article are available online at http://microbialcell.com/researcharticles/2026a-olgun-microbial-cell/.

.

## CONFLICT OF INTEREST

The authors declare no conflict of interest.

## ABBREVIATION

AMA – Antimycin A

DEG – Differentially Expressed Gene

E2 – 17β-Estradiol

ER – Estrogen Receptor

FTIR – Fourier Transform Infrared Spectroscopy

GO – Gene Ontology

H_2_DCFDA – 2′,7′-Dichlorodihydrofluorescein Diacetate

HCA – Hierarchical Cluster Analysis

*moh1*Δ – MOH1 Deleted

PCA – Principal Component Analysis

ROS – Reactive Oxygen Species

RNA-Seq – RNA Sequencing

RT-qPCR – Reverse Transcription Quantitative Polymerase Chain Reaction

SEM – Scanning Electron Microscopy

SGD – Saccharomyces Genome Database

WB – Western Blot

WT – Wild Type

YPD – Yeast Extract Peptone Dextrose

YPEL – Yippee-Like Protein

YPRG – Yeast Extract Peptone Raffinose Galactose
