# Supplementary material for: Yippee-like protein Moh1 links gene expression to metabolism and selective stress resistance in Saccharomyces cerevisiae
Source: Microb Cell. 2026 Jun 29;13:261–81. doi: 10.15698/mic2026.06.881 (PMC13329494; doi:10.15698/mic2026.06.881)
Supplement: Supplementary file 1 — . [file mic-13-261-s01.pdf]

SUPPLEMENTARY INFORMATION

Yippee-like protein Moh1 links gene expression to metabolism and selective stress resistance in *Saccharomyces cerevisiae*

Çağla Ece Olgun[1*], Gizem Turan Duman[1*], Gizem Güpür[1], Hamit İzgi[1,] Mariam Huda[2], Demet Çetin[3], Zekiye Suludere[4], Fatma Küçük Baloğlu[5], Ayşe Koca Çaydaşı[2, 6], Mesut Muyan[1, 6]

[1]Department of Biological Sciences, Middle East Technical University, 06800 Çankaya-Ankara, Türkiye

[2]Department of Molecular Biology and Genetics, Koç University, Istanbul, Türkiye

[3]Department of Mathematics and Science Education, Gazi Faculty of Education, Gazi University, 06500 Ankara, Türkiye

[4]Department of Biology, Faculty of Science, Gazi University, 06500 Ankara, Türkiye

[5]Department of Biology, Giresun University, Giresun, Türkiye

[6]Correspondence: aykoca@ku.edu.tr & mmuyan@metu.edu.tr

* Equal contribution: Should be considered as the first author

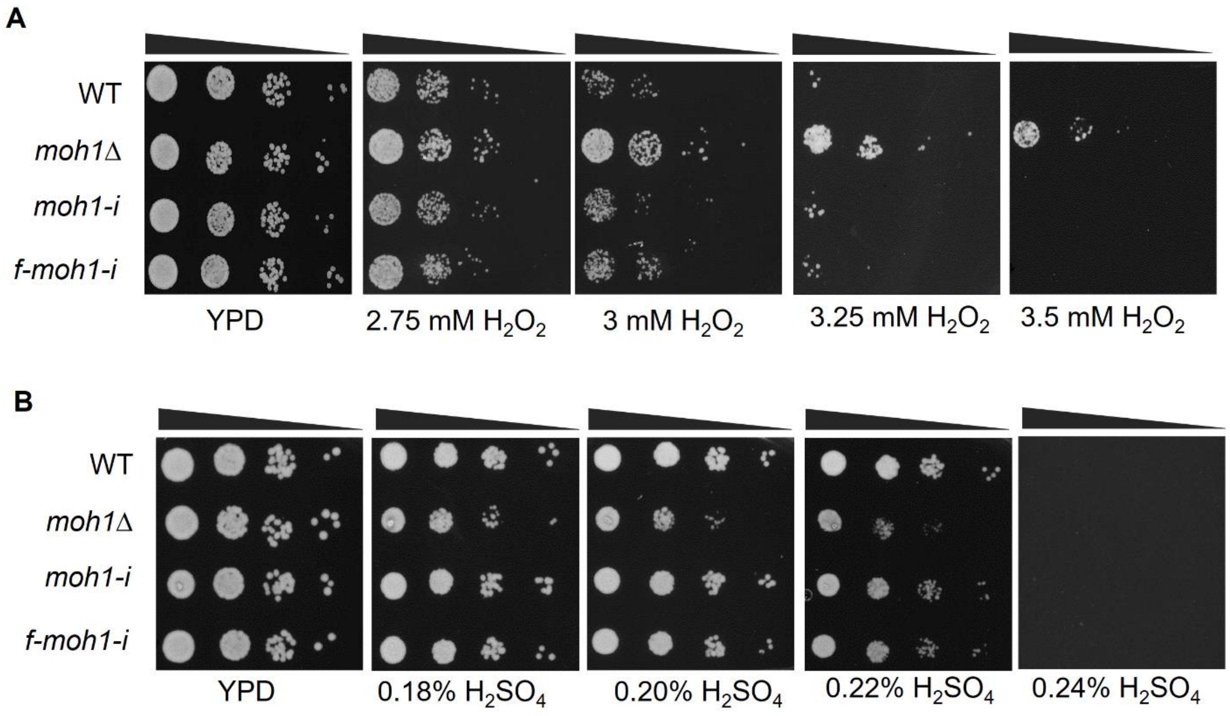

Figure S1. Assessing the effects of stressor concentrations on yeast strains. (A & B) WT, *moh1*Δ, *moh1*Δ*-i,* and *f-moh1-i* cells from subcultures were grown until OD_{600} of 0.4-0.6. Cells, 2.5 x 10^6 cells/mL, with 10-fold serial dilutions (black triangles), were then spotted on (**A**) the YPD-Agar plate containing none (YPD) or 2.75, 3.00, 3.25, or 3.50 mM hydrogen peroxide (H_2O_2), or (**B**) 0.18, 0.20, 0.22, or 0.24 % sulfuric acid (H_2SO_4). Plates were incubated at 30°C for 40 hours and photographed.

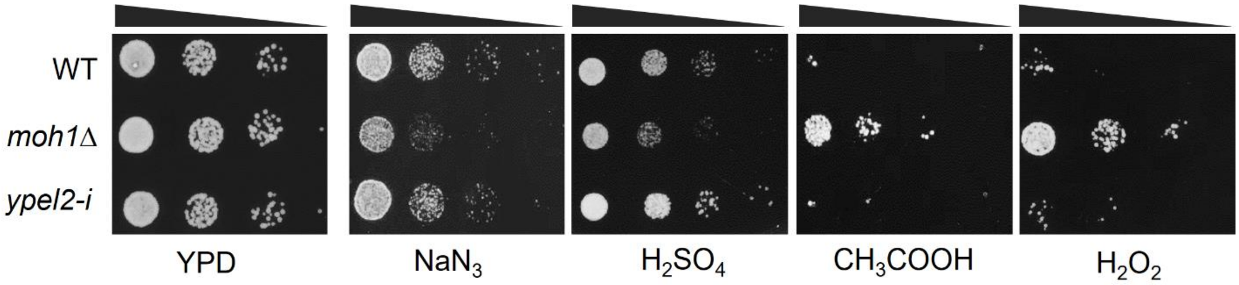

Figure S2. YPEL2 complements Moh1p in spot tests without or with a stressor. For growth on water, single colonies of WT, *moh1*Δ, and ypel2-i cells were grown overnight in YPD, sub-cultured 1:100 into fresh YPD, and incubated for one week. After washing and resuspension in sterile water, cells were incubated at 30 °C with shaking. On day 14, cultures were serially diluted and spotted on agar plates, then incubated at 30 °C for 40 hours before imaging. For other stress inducers, WT, *moh1*Δ, and *ypel2-i* cells from subcultures were grown until OD_{600} of 0.4-0.6. 2.5 x 10^6 cells/mL were then spotted on the YPD-Agar plate containing none (YPD) or 0.4 mM NaN_3, 0.22% H_2SO_4, 40 mM CH_3COOH, and 3.25 mM H_2O_2 with 10-fold serial dilutions. Plates were incubated at 30°C for 40 hours and photographed.

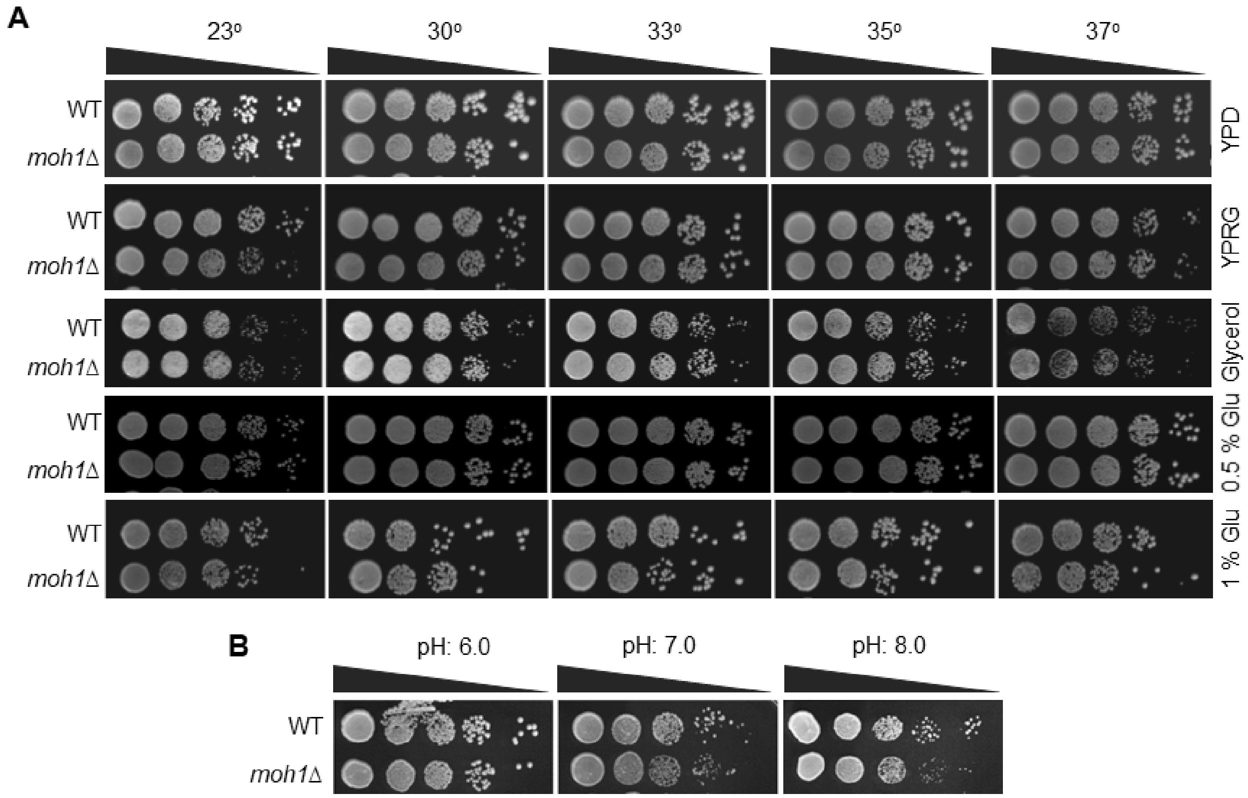

Figure S3. Assessing the effects of nutritional adjustments under different temperature or pH on WT and *moh1Δ* cells. (A) WT and *moh1*Δ cells from subcultures were grown until OD_{600} of 0.4-0.6 in YPD. $2.5x10^6$ cells/mL was used for 10-fold serial dilutions for spotting onto the indicated agar plates: YPD (1% Yeast extract, 2% Peptone, 2% Dextrose), YPRG (1% Yeast extract, 2% Peptone, 3% Raffinose, and 2% Galactose), Glycerol (1% Yeast extract, 2% Peptone, 3% Glycerol), 0.5% Glucose (1% Yeast extract, 2% Peptone, 0.5% Glucose), 1% Glucose (1% Yeast extract, 2% Peptone, 1% Glucose). Plates were incubated at 23, 30, 33, 35, 37 °C for 40 hours and photographed. (**B**) For spot tests at different pH, buffered YPD agar plates prepared YPD-agar base (1% yeast extract, 2% peptone, 2% dextrose and 2% agar), sterilized by autoclaving. The molten medium was then supplemented with sterile buffer to the indicated pH while maintaining a constant final buffer strength (~50 mM) across conditions. For pH 6-8, a citrate-phosphate system was used by combining citric acid with Na_2HPO_4 at pH-specific ratios to achieve the target pH. Plates were incubated at 30°C for 40 hours and photographed.

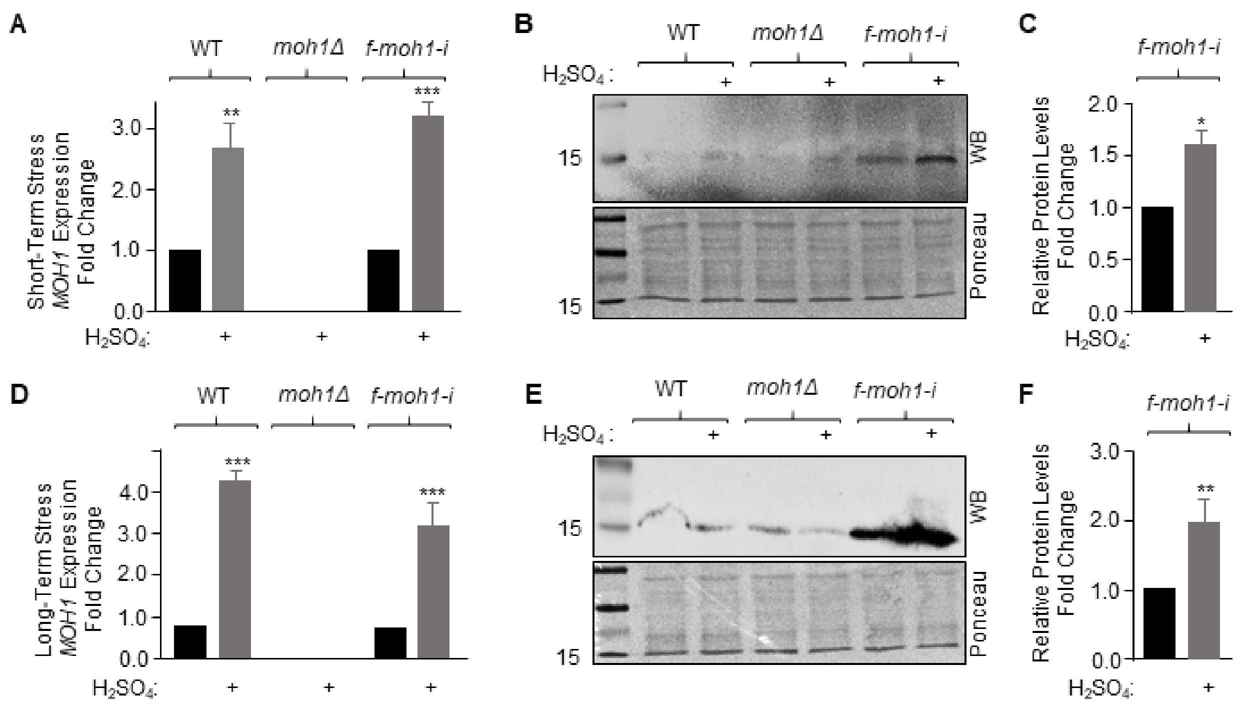

Figure S4. Effects of H_2SO_4 on Moh1 levels. The expression (**A & D**) and synthesis (**B-F**) of f-Moh1 in WT, *moh1*Δ, and *f-moh1-i* cells was assessed with WB using the Flag antibody after H_2SO_4 exposure for a short term of 45 min **(A-C)** or a long term of 40 h (**D-F**). Expression of MOH1 using primers specific to MOH1 was normalized to transcript levels of *YPR062W (FCY1*) and *YNL219C (ALG9)* as internal controls in RT-qPCR. In WB analysis, the level of Flag-Moh1 migrating at ~16 kDa in the absence of H_2O_2 was set to one and compared to that observed in the presence of H_2O_2. A band of similar molecular mass was also detected in both WT and *moh1Δ* strains, indicating that it represents a non-specific signal. Ponceau staining was used as a control for equal loading in WB. Molecular weight markers in kDa are indicated. ***, ** and * indicate p<0.001, p<0.01, and p<0.05, respectively.

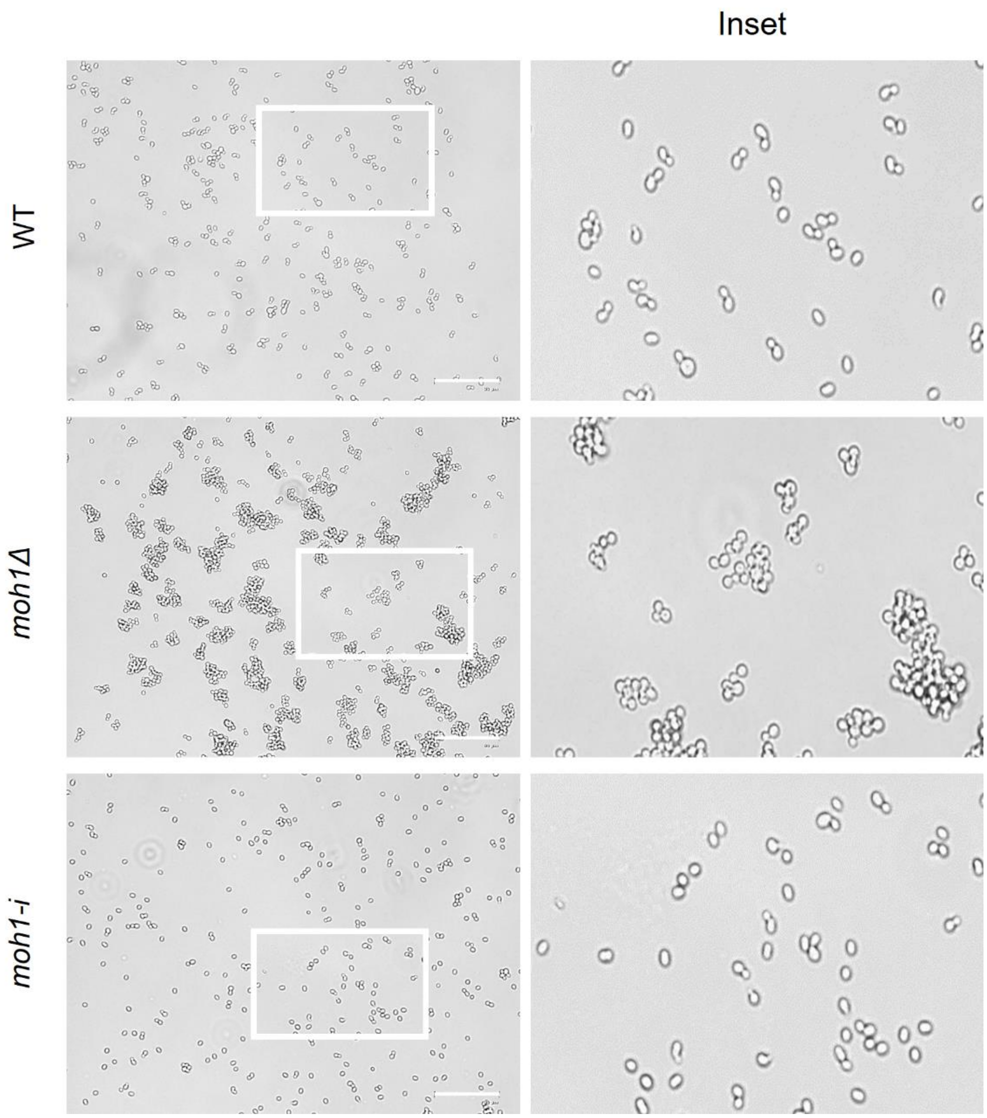

Figure S5. Light microscopy images of yeast strains. WT, *moh1*Δ, and *moh1-i* cells from subcultures were plated on coverslips and visualized with a light microscope. White squares indicate the Inset. Scale bars are shown.

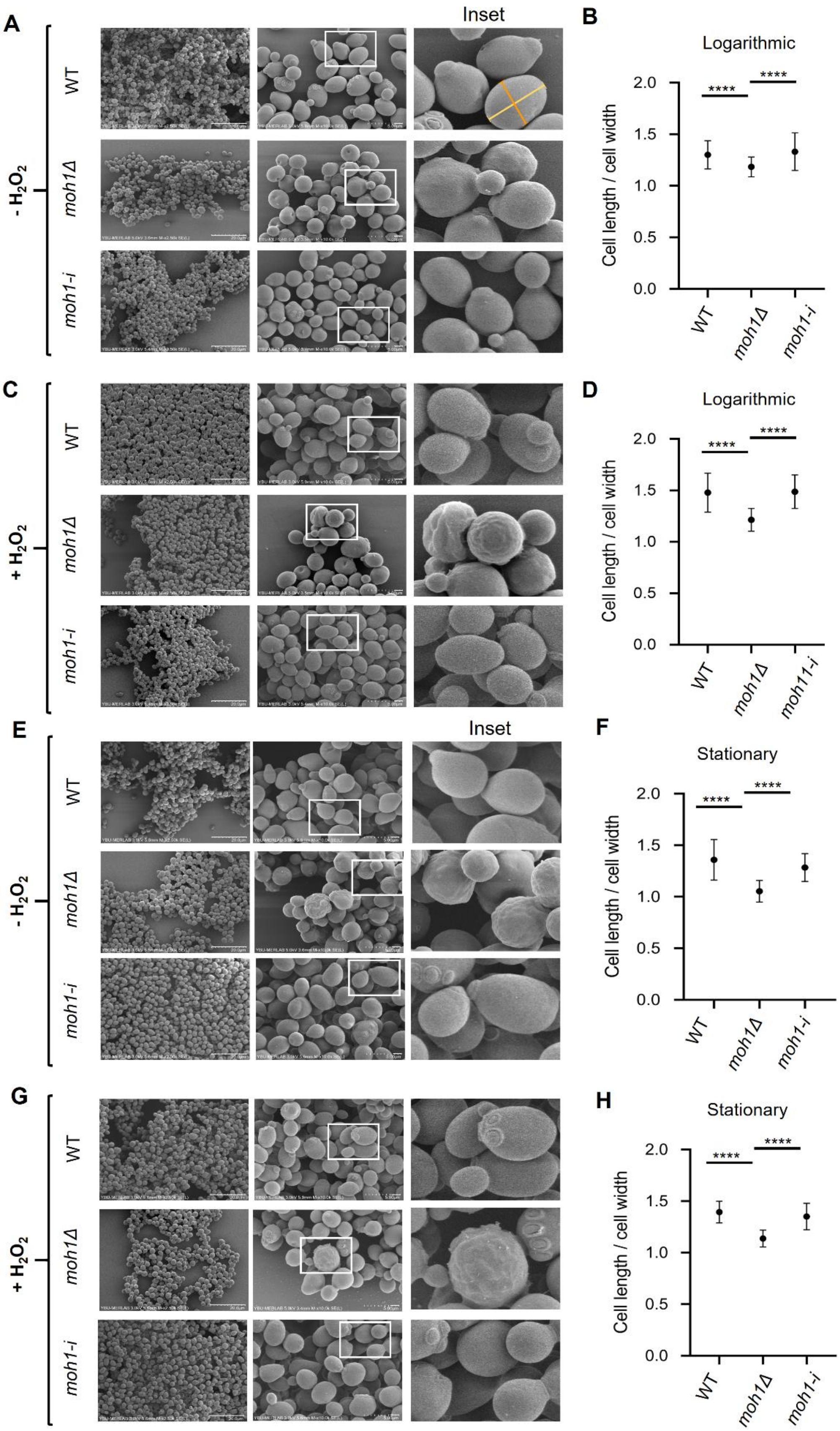
A
B
C
D
E
F
G
H
Inset
Inset
- H_2O_2
+ H_2O_2
- H_2O_2
+ H_2O_2
WT
moh1Δ
moh1-i
Logarithmic
Logarithmic
Stationary
Stationary
Cell length / cell width
0.0
0.5
1.0
1.5
2.0
****
WT
moh1Δ
moh1-i
moh11-i

Figure S6. SEM of yeast strains grown on agar, logarithmic or stationary phase, in the absence or presence of H_2O_2 as a stressor. (**A-H**) A single colony of WT, *moh1*Δ, and *moh1-i* cells grown on YPD-Agar plates was inoculated into YPD medium and incubated overnight at 30 °C with shaking at 180 rpm. To obtain cells in the logarithmic phase (**A-D**), cultures were diluted 1:100 and grown at 30 °C with shaking at 180 rpm until OD_{600} of 0.4–0.6. For the stationary phase (**E-H**), cells were grown for 48 hours at 30 °C with reciprocal shaking at 180 rpm. (**A-H**) Cells in logarithmic or stationary phase were then subjected to none or 3.25 mM H_2O_2 for 45 min as short-term stress. Cells were collected and centrifuged at 1000 rpm for 3 minutes. Cell pellets were resuspended in 4% glutaraldehyde for fixation, followed by dehydration with an ascending series of ethanol and air-drying. Samples were coated with gold and imaged using a Scanning Electron Microscope (SEM). White squares indicate insets. Scale bars are shown (**B & D, F & H**). The cell length and width (indicated with solid lines) ratio of yeast strains in the absence (**B & F**) or the presence (**D & H**) of H_2O_2 was graphed using 50 cells from images. A Student's t-test was conducted for statistical analyses. **** indicates <0.001.

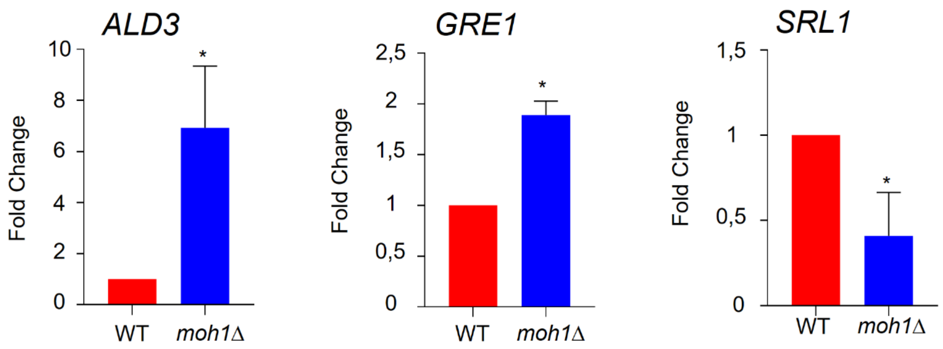

Figure S7. Verification of RNA-Seq results via RT-qPCR. Expression of *ALD3*, *GRE1*, and *SRL1* was assessed by RT-qPCR using RNA samples prepared for RNA-Seq. Results normalized to the geometric means of *YPR062W* (*FCY1*) and *YNL219C* (*ALG9*) expressions as the internal control are presented as the mean ± S.E.M. with a Student's t-test for the statistical significance, *p <0.05.

SUPPLEMENTARY INFORMATION

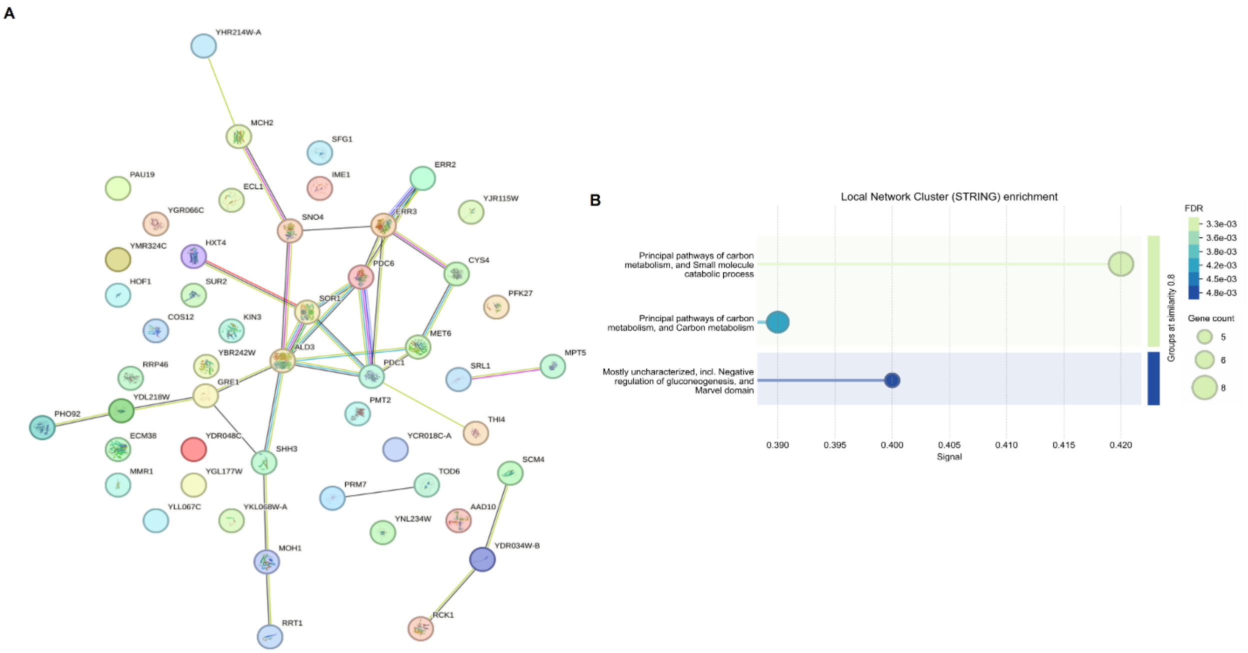

Figure S8. Analysis of DEGs by STRING. (**A**) Protein network of DEGs (**B**) Local network Cluster enrichment of DEGs.

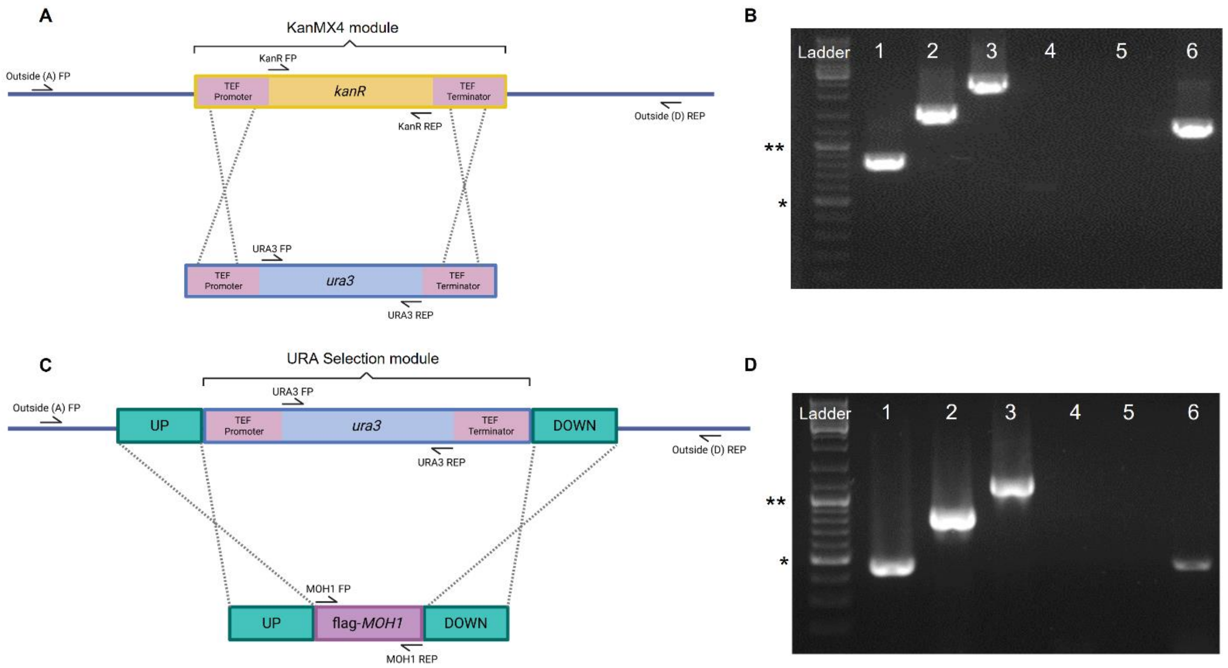

Figure S9. Assessing the genomic insertion of *URA3* or *MOH1* with PCR. (**A**) Schematic representation of homologous recombination between KanR and URA3 selection module in *moh1Δ::KanMX4* cells and primer binding sites. (**B**) Following the insertion of the URA3-selection module into the *moh1Δ::KanMX4* strain, single colonies grown in SC-URA selective medium were used to screen transformants. The genomic DNA from cells was used as the template for PCR using primer sets specific for Lane 1: PCR with URA3 FP and URA3 REP; Lane 2: PCR with URA3 FP and Outside (D) REP; Lane 3: Outside (A) FP and Outside (D) REP Lane 4: PCR with KanR FP and KanR REP; Lane 5: No template control; Lane 6: Positive control (T7 FP and T3 REP primers, p426GPD plasmid as template). (**C**) Schematic representation of homologous recombination between the *URA3* selection module and *flag-MOH1* module in *moh1Δ::URA3* cells and primer binding sites. (**D**) For screening of the *flag-MOH1* module inserted *moh1Δ::URA3* strain, we used PCR with primers specific for Lane 1: MOH1 FP and MOH1 REP; Lane 2: MOH1 FP and outside (D) REP; Lane 3: Outside (A) FP and Outside (D) REP; Lane 4: URA3 FP & URA3 REP; Lane 5: No template control; Lane 7: Positive control (MOH1 FP and MOH1 REP; WT-BY4741 gDNA as template). The DNA ladder is indicated: *: 500 bp, **: 1000 bp.

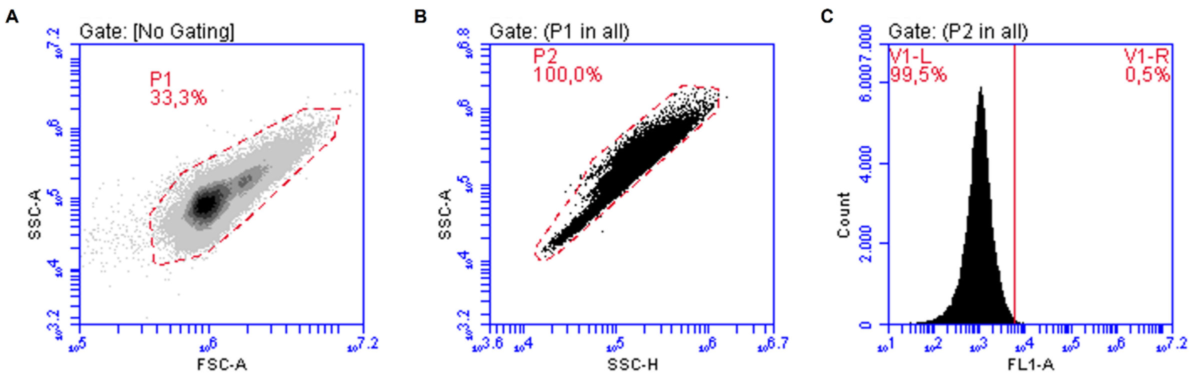

Figure S10. The gating strategy for flow cytometry. All gating parameters were established based on WT control cells that were neither exposed to stress conditions nor treated with H_2DCFDA. **(A)** The total cell population is gated on a dot plot of forward scatter area (FSC-A) versus side scatter area (SSC-A), resulting in the selection of a child population labeled P1. **(B)** Single cells were gated from population P1 using a dot plot of side scatter height (SSC-H) versus side scatter area (SSC-A), resulting in population P2. **(C)** Fluorescence intensity was assessed within the P2 population following stress and H_2DCFDA treatments to determine the percentage distribution of fluorescent cells.

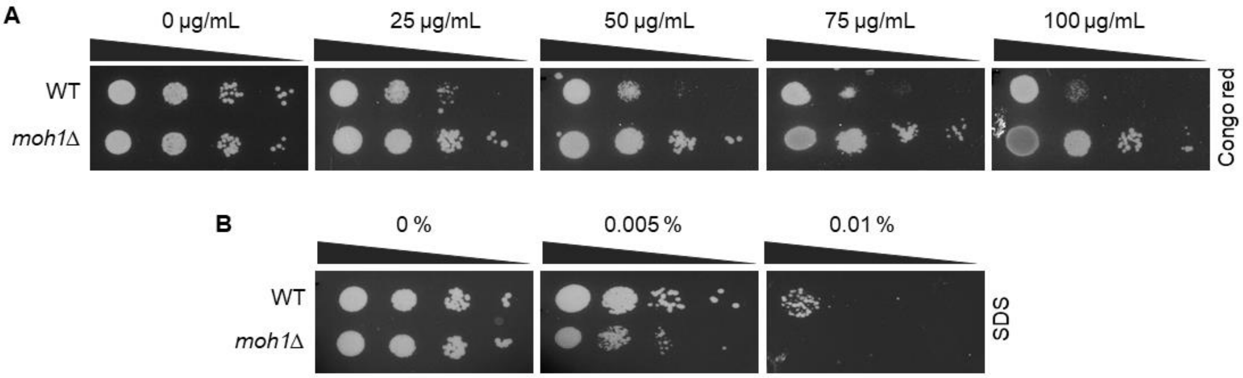

Figure S11. Effects of Congo red and SDS on WT and *moh1*Δ strains. WT and *moh1*Δ cells from subcultures were grown until OD_{600} of 0.4-0.6. Cells, 2.5 x 10^6 cells/mL, with 10-fold serial dilutions (black triangles), were then spotted on (**A**) the YPD-Agar plate containing none (0), 25, 50, 75 or 100 µg/mL Congo red, or (**B**) none (0), 0.005 or 0.01% SDS. Plates were incubated at 30°C for 40 hours and photographed.

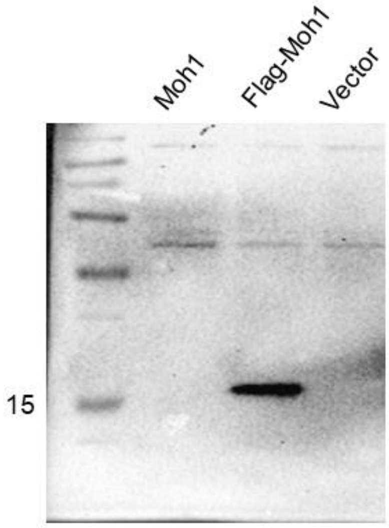

Figure S12. Assessing the specific recognition of Flag-Moh1 by the Flag antibody. COS7 cells were transiently transfected with the expression vector bearing none, MOH1 or Flag-MOH1 cDNA. Twenty-four hours after transfections, cells were collected, and equal amounts (50 µg) of protein extracts were subjected to SDS-10% PAGE followed by WB analysis using the Flag antibody. Molecular mass is indicated in kDa.

Supplementary Information Table 1. Phyre2

Moh1 Phyre2 Results (Confidence>80)

#	Template	Confidence	id %	Template Information
1	c8qbnY_	100	37	**PDB header:** ligase<br>**Chain:** Y<br>**PDB Molecule:** protein yippee-like 5<br>**PDBTitle:** structure of the non-canonical ctlh e3 substrate receptor wdr26 bound to ypel5
2	c7sfzC_	96.9	22	**PDB header:** cell cycle<br>**Chain:** C<br>**PDB Molecule:** protein mis18-alpha<br>**PDBTitle:** crystal structure of mis18a-yippee domain
3	c6uml C_	97.5	25	**PDB header:** ligase<br>**Chain:** C<br>**PDB Molecule:** protein cereblon<br>**PDBTitle:** structural basis for thalidomide teratogenicity revealed by the2 cereblon-ddb1-sall 4-pomalidomide complex
4	c2k8dA_	90.7	20	**PDB header:** oxidoreductase<br>**Chain:** A<br>**PDB Molecule:** peptide methionine sulfoxide reductase msrb<br>**PDBTitle:** solution structure of a zinc-binding methionine sulfoxide reductase
5	c4v30A_	97.2	21	**PDB header:** signaling protein<br>**Chain:** A<br>**PDB Molecule:** cereblon isoform 4<br>**PDBTitle:** cereblon isoform 4 from magnetospirillum gryphiswaldense in complex2 with lenalidomide
6	c2l1uA_	88.3	17	**PDB header:** oxidoreductase<br>**Chain:** A<br>**PDB Molecule:** methionine-r-sulfoxide reductase b2, mitochondrial<br>**PDBTitle:** structure-functional analysis of mammalian msrb2 protein
7	c6m6rA_	95.5	20	**PDB header:** rna binding protein/rna<br>**Chain:** A<br>**PDB Molecule:** dicer related helicase<br>**PDBTitle:** crystal structure of caenorhabditis elegans dicer- related helicase 32 (drh-3) c-terminal domain with 5'-ppp 8-mer ssrna
8	c4a2vA_	88.9	18	**PDB header:** hydrolase<br>**Chain:** A<br>**PDB Molecule:** retinoic acid inducible protein i<br>**PDBTitle:** structure of duck rig-i c-terminal domain (ctd)
9	c3hcj B_	90.8	16	**PDB header:** oxidoreductase<br>**Chain:** B<br>**PDB Molecule:** peptide methionine sulfoxide reductase<br>**PDBTitle:** structure of msrb from xanthomonas campestris (oxidized2 form)
10	c3ga3A_	95.5	19	**PDB header:** hydrolase<br>**Chain:** A<br>**PDB Molecule:** interferon-induced helicase c domain- containing protein 1<br>**PDBTitle:** crystal structure of the c-terminal domain of human mda5
11	c2w4rB_	95.1	16	**PDB header:** hydrolase<br>**Chain:** B<br>**PDB Molecule:** probable atp-dependent rna helicase dhx58;<br>**PDBTitle:** crystal structure of the regulatory domain of human lgp2

Supplementary Information Table 1. Phyre2

12	c6symB_	89.6	16	**PDB header:** hydrolase<br>**Chain:** G<br>**PDB Molecule:** probable atp-dependent rna helicase ddx58;<br>**PDBTitle:** crystal structure of the regulatory domain of human rig- i with bound2 zn
13	c2qfbG_	84.7	16	**PDB header:** hydrolase<br>**Chain:** G<br>**PDB Molecule:** probable atp-dependent rna helicase ddx58<br>**PDBTitle:** crystal structure of the regulatory domain of human rig- i with bound2 Zn
14	c2kaoA_	91.6	22	**PDB header:** oxidoreductase<br>**Chain:** A<br>**PDB Molecule:** methionine-r-sulfoxide reductase b1<br>**PDBTitle:** structure of reduced mouse methionine sulfoxide reductase b12 (sec95cys mutant)
15	c6tr8A_	90.3	15	**PDB header:** oxidoreductase<br>**Chain:** A<br>**PDB Molecule:** peptide-methionine (r)-s-oxide reductase;<br>**PDBTitle:** corynebacterium diphtheriae methionine sulfoxide reductase b (msrb)2 solution structure - reduced form
16	c5hj0C_	97.1	14	**PDB header:** ligase<br>**Chain:** C<br>**PDB Molecule:** kinetochore protein mis18<br>**PDBTitle:** crystal structure of mis18 ' yippee-like' domain
17	c7e43A_	86.6	15	**PDB header:** oxidoreductase<br>**Chain:** A<br>**PDB Molecule:** peptide methionine sulfoxide reductase msra/msrb<br>**PDBTitle:** structural insights into a bifunctional peptide methionine sulfoxide2 reductase msra/b fusion protein from helicobacter pylori
18	c3cezA_	92.8	16	**PDB header:** oxidoreductase<br>**Chain:** A<br>**PDB Molecule:** methionine-r-sulfoxide reductase<br>**PDBTitle:** crystal structure of methionine-r-sulfoxide reductase from2 burkholderia pseudomallei
19	c5fa9B_	91.8	13	**PDB header:** oxidoreductase<br>**Chain:** B<br>**PDB Molecule:** peptide methionine sulfoxide reductase msra<br>**PDBTitle:** bifunctional methionine sulfoxide reductase ab (msrab) from treponema2 denticola
20	c3lrrB_	85.6	17	**PDB header:** hydrolase/rna<br>**Chain:** B<br>**PDB Molecule:** probable atp-dependent rna helicase ddx58<br>**PDBTitle:** crystal structure of human rig-i ctd bound to a 12 bp au rich 5' ppp2 dsrna
21	c6qa0A_	90.3	11	**PDB header:** oxidoreductase<br>**Chain:** A<br>**PDB Molecule:** methionine-r-sulfoxide reductase b3;<br>**PDBTitle:** msrb3 - aa 1-137
22	c5e24D_	89.4	64	**PDB header:** transport/dna binding/dna<br>**Chain:** D<br>**PDB Molecule:** protein hairless;<br>**PDBTitle:** structure of the su(h)-hairless-dna repressor complex
23	d1xm0a1	89.4	12	**Fold:** Mss4-like<br>**Superfamily:** Mss4-like<br>**Family:** Sel R domain

Supplementary Information Table 1. Phyre2

24	d1l1da_	89.7	11	**Fold:** Mss4-like<br>**Superfamily:** Mss4-like<br>**Family:** Sel R domain
25	c7ctoF_	94	7	**PDB header:** oxidoreductase<br>**Chain:** F<br>**PDB Molecule:** peptide methionine sulfoxide reductase msrb<br>**PDBTitle:** staphylococcus aureus msrb
26	c3e0mB_	93.3	7	**PDB header:** oxidoreductase<br>**Chain:** B<br>**PDB Molecule:** peptide methionine sulfoxide reductase msra/msrb 1<br>**PDBTitle:** crystal structure of fusion protein of msra and msrb

YPEL2 Phyre2 Results (Confidence>80)

#	Template	Confidence	id %	Template Information
1	c8qbnY_	100	45	**PDB header:** ligase<br>**Chain:** Y<br>**PDB Molecule:** protein yippee-like 5<br>**PDBTitle:** structure of the non-canonical ctlh e3 substrate receptor wdr26 bound2 to ypel 5
2	c2k8dA_	93.6	21	**PDB header:** oxidoreductase<br>**Chain:** A<br>**PDB Molecule:** peptide methionine sulfoxide reductase msrb<br>**PDBTitle:** solution structure of a zinc-binding methionine sulfoxide reductase
3	c4v30A_	97.4	24	**PDB header:** signaling protein<br>**Chain:** A<br>**PDB Molecule:** cereblon isoform 4<br>**PDBTitle:** cereblon isoform 4 from magnetospirillum gryphiswaldense in complex2 with lenalidomide
4	c6umlC_	97.6	22	**PDB header:** ligase<br>**Chain:** C<br>**PDB Molecule:** protein cereblon<br>**PDBTitle:** structural basis for thalidomide teratogenicity revealed by the2 cereblon-ddb1-sall 4-pomalidomide complex
5	c2w4rB_	92.5	19	**PDB header:** hydrolase<br>**Chain:** B<br>**PDB Molecule:** probable atp-dependent rna helicase dhx58<br>**PDBTitle:** crystal structure of the regulatory domain of human lgp2
6	c6qa0A_	94.1	18	**PDB header:** oxidoreductase<br>**Chain:** A<br>**PDB Molecule:** methionine-r-sulfoxide reductase b3<br>**PDBTitle:** msrb3 - aa 1-137
7	c6tr8A_	93.9	21	**PDB header:** oxidoreductase<br>**Chain:** A<br>**PDB Molecule:** peptide-methionine (r)-s-oxide reductase<br>**PDBTitle:** corynebacterium diphtheriae methionine sulfoxide reductase b (msrb)2 solution structure - reduced form

Supplementary Information Table 1. Phyre2

8	c3lrrB_	85.5	19	**PDB header:** hydrolase/rna<br>**Chain:** B<br>**PDB Molecule:** probable atp-dependent rna helicase ddx58<br>**PDBTitle:** crystal structure of human rig-i ctd bound to a 12 bp au rich 5' ppp2 dsrna
9	c6symB_	94	19	**PDB header:** oxidoreductase<br>**Chain:** B<br>**PDB Molecule:** peptide methionine sulfoxide reductase msrb<br>**PDBTitle:** crystal structure of escherichia coli msrb (reduced form)
10	c4a2vA_	90	16	**PDB header:** hydrolase<br>**Chain:** A<br>**PDB Molecule:** retinoic acid inducible protein i<br>**PDBTitle:** structure of duck rig-i c-terminal domain (ctd)
11	c6m6rA_	94.8	21	**PDB header:** rna binding protein/rna<br>**Chain:** A<br>**PDB Molecule:** dicer related helicase<br>**PDBTitle:** crystal structure of caenorhabditis elegans dicer- related helicase 32 (drh-3) c-terminal domain with 5'-ppp 8-mer ssrna
12	c3hcjB_	93.2	15	**PDB header:** oxidoreductase<br>**Chain:** B<br>**PDB Molecule:** peptide methionine sulfoxide reductase<br>**PDBTitle:** structure of msrb from xanthomonas campestris (oxidized2 form)
13	c7sfzC_	97	16	**PDB header:** cell cycle<br>**Chain:** C<br>**PDB Molecule:** protein mis18-alpha<br>**PDBTitle:** crystal structure of mis18a-yippee domain
14	c2qfbG_	88.6	15	**PDB header:** hydrolase<br>**Chain:** G<br>**PDB Molecule:** probable atp-dependent rna helicase ddx58<br>**PDBTitle:** crystal structure of the regulatory domain of human rig- i with bound Zn
15	c5hj0C_	97.1	13	**PDB header:** ligase<br>**Chain:** C<br>**PDB Molecule:** kinetochore protein mis18<br>**PDBTitle:** crystal structure of mis18 ' yippee-like' domain
16	c5fa9B_	94.8	13	**PDB header:** oxidoreductase<br>**Chain:** B<br>**PDB Molecule:** peptide methionine sulfoxide reductase msra<br>**PDBTitle:** bifunctional methionine sulfoxide reductase ab (msrab) from treponema2 denticola
17	c2l1uA_	92.1	15	**PDB header:** oxidoreductase<br>**Chain:** A<br>**PDB Molecule:** methionine-r-sulfoxide reductase b2, mitochondrial<br>**PDBTitle:** structure-functional analysis of mammalian msrb2 protein
18	c2kaoA_	90.3	20	**PDB header:** oxidoreductase<br>**Chain:** A<br>**PDB Molecule:** methionine-r-sulfoxide reductase b1<br>**PDBTitle:** structure of reduced mouse methionine sulfoxide reductase b12 (sec95cys mutant)

Supplementary Information Table 1. Phyre2

19	c7ctoF_	95.3	12	**PDB header:** oxidoreductase<br>**Chain:** F<br>**PDB Molecule:** peptide methionine sulfoxide reductase msrb<br>**PDBTitle:** staphylococcus aureus msrb
20	c3ga3A_	91	13	**PDB header:** hydrolase<br>**Chain:** A<br>**PDB Molecule:** interferon-induced helicase c domain-containing protein 1<br>**PDBTitle:** crystal structure of the c-terminal domain of human mda5
21	c3cezA_	94.7	14	**PDB header:** oxidoreductase<br>**Chain:** A<br>**PDB Molecule:** methionine-r-sulfoxide reductase<br>**PDBTitle:** crystal structure of methionine-r-sulfoxide reductase from2 burkholderia pseudomallei
22	c3e0mB_	94.7	11	**PDB header:** oxidoreductase<br>**Chain:** B<br>**PDB Molecule:** peptide methionine sulfoxide reductase msra/msrb 1<br>**PDBTitle:** crystal structure of fusion protein of msra and msrb
23	d1xm0a1	92.3	12	**Fold:** Mss4-like<br>**Superfamily:** Mss4-like<br>**Family:** Sel R domain
24	c7e43A_	92	12	**PDB header:** oxidoreductase<br>**Chain:** A<br>**PDB Molecule:** peptide methionine sulfoxide reductase msra/msrb<br>**PDBTitle:** structural insights into a bifunctional peptide methionine sulfoxide2 reductase msra/b fusion protein from helicobacter pylori
25	c5e24D_	88.2	57	**PDB header:** transport/dna binding/dna<br>**Chain:** D<br>**PDB Molecule:** protein hairless;<br>**PDBTitle:** structure of the su(h)-hairless-dna repressor complex
26	d1l1da_	94.3	8	**Fold:** Mss4-like<br>**Superfamily:** Mss4-like<br>**Family:** Sel R domain

Supplementary Information Table S2. Differentially Expressed Genes

Systematic Name	Gene Names	log2(FC)	Adjusted p-value	Description	Examples of conditions in which gene expression changes	References
YDR048C	YDR048C	4.8869774	0.04198109	Dubious open reading frame; unlikely to encode a functional protein, based on available experimental and comparative sequence data; partially overlaps ORF VMS1/YDR049W	osmotic stress, oxidative stress, metal or metalloid ion stress	[156-158]
YMR323W	ERR3	3.9636335	4.25952E-19	Enolase, a phosphopyruvate hydratase; catalyzes the conversion of 2-phosphoglycerate to phosphoenolpyruvate; complements the growth defect of an ENO1 ENO2 double mutant in glucose	heat shock stress, osmotic stress, metal or metalloid iron stress	[159-161]
YMR324C	YMR324C	3.9309242	0.003093842	Dubious open reading frame; unlikely to encode a functional protein, based on available experimental and comparative sequence data; transcription is AZF1 dependent in glycerol-lactate medium and SLT2 dependent in response to the lipid hydroperoxides	osmotic stress, oxidative stress	[156, 161]
YMR325W	PAU19	3.5718044	0.000279455	Protein of unknown function; member of the seripauperin multigene family encoded mainly in subtelomeric regions	oxidative stress, oxidative stress	[162]
YDL218W	YDL218W	2.6161261	2.38944E-06	Putative protein of unknown function; YDL218W transcription is regulated by Azf1p and induced by starvation and aerobic conditions; expression also induced in cells treated with the mycotoxin patulin	osmotic stress, oxidative stress	[156, 161, 162]
YPL281C	ERR2	2.1497823	0.029049994	Enolase, a phosphopyruvate hydratase; catalyzes the conversion of 2-phosphoglycerate to phosphoenolpyruvate; complements the growth defect of an ENO1 ENO2 double mutant	osmotic stress, metal or metalloid iron stress	[159, 163,164]
YDR374C	PHO92	2.1227533	0.01028738	N6-Methyladenosine (m6A) reader; co-transcriptionally recruited to specific methylated mRNAs during meiotic prophase, facilitating protein synthesis and subsequent decay of m6A modified transcripts, resulting in timely meiotic recombination; regulates PHO4 mRNA stability, binding to the 3'UTR in a phosphate-dependent manner; posttranscriptional regulator of phosphate and glucose metabolism; contains a conserved YTH domain with RNA-binding activity; human homolog YTHDF2 complements a null mutant	DNA damage stress, metal or metalloid iron stress, oxidative stress	[165-167]
YDL039C	PRM7	2.0727062	0.002499803	Pheromone-regulated protein; predicted to have one transmembrane segment; promoter contains Gcn4p binding elements; in W303 strain one continuous open reading frame comprising of YDL037C, the intergenic region and YDL039C encodes the IMI1	osmotic stress, metal or metalloid iron stress, oxidative stress	[159, 163, 168]
YDR034W-B	CPP3	1.8332894	4.57325E-06	Tail-anchored plasma membrane (PM) protein; C-terminal palmitoylation is required for membrane anchoring and protein stability; PM localization is polarized to the daughter cell; also localizes to the vacuolar membrane; may be involved in response to	DNA damage stress, osmotic stress	[158]

Supplementary Information Table S2. Differentially Expressed Genes

				stress; upregulated by toxic concentrations of heavy metal ions and alkali; contains a conserved cysteine rich palmitoylated domain (CYSPD, aka CYSTM); CPP3 has a paralog, MNC1, that arose from the whole genome duplication		
YHR092C	HXT4	1.7773507	1.02333E-08	High-affinity glucose transporter; member of the major facilitator superfamily, expression is induced by low levels of glucose and repressed by high levels of glucose; HXT4 has a paralog, HXT7, that arose from the whole genome duplication	osmotic stress, DNA damage stress	[163, 165, 169]
YGR087C	PDC6	1.7412796	0.00036334	Minor isoform of pyruvate decarboxylase; decarboxylates pyruvate to acetaldehyde, involved in amino acid catabolism; transcription is glucose- and ethanol-dependent, and is strongly induced during sulfur limitation	osmotic stress, DNA damage stress, metal or metalloid iron stress	[159, 163, 165]
YJR155W	AAD10	1.5665967	2.37614E-05	Putative aryl-alcohol dehydrogenase; similar to P. chrysosporium aryl-alcohol dehydrogenase; mutational analysis has not yet revealed a physiological role; members of the AAD gene family comprise three pairs (AAD3 + AAD15, AAD6/AAD16 + AAD4, AAD10 + AAD14) whose two genes are more related to one another than to other members of the family	osmotic stress, metal or metalloid iron stress, oxidative stress	[157, 163,166, 171]
YJR094C	IME1	1.5453213	0.004113023	Master meiotic regulator active only during meiotic events; activates transcription of early meiotic genes through interaction with Ume6p; regulator of meiotic commitment; phosphorylated by Rim11p; degraded by the 26S proteasome following phosphorylation by Ime2p; transcription is negatively regulated in cis by the IRT1 long noncoding antisense RNA	osmotic stress, metal or metalloid iron stress, oxidative stress	[159, 163, 168]
YNCJ0028C	IRT1	1.4949028	5.73589E-06	Long noncoding RNA that governs mating-type control of gametogenesis; located in the IME1 promoter; in haploids, expression of IME1, the central inducer of gametogenesis, is inhibited in cis by transcription of IRT1, which recruits the Set2p histone methyltransferase and the Set3p histone deacetylase complex to establish repressive chromatin at the IME1 promoter		
YGL158W	RCK1	1.458466	0.047126492	Protein kinase involved in oxidative stress response; promotes pseudohyphal growth via activation of Ubp3p phosphorylation; identified as suppressor of S. pombe cell cycle checkpoint mutations; RCK1 has a paralog, RCK2, that arose from the whole genome duplication	heat shock, osmotic stress	[160, 161]
YMR322C	SNO4	1.3293445	0.02199161	Possible chaperone and cysteine protease; required for transcriptional reprogramming during the diauxic shift and for survival in stationary phase; similar to bacterial Hsp31 and yeast Hsp31p, Hsp32p, and Hsp33p; DJ-1/ThiJ/PfpI superfamily member; predicted involvement in pyridoxine metabolism; induced by mild heat stress and copper deprivation	DNA damage stress, metal or metalloid iron stress	[159, 165, 169, 172]
YGR066C	GID10	1.3191628	0.031497663	Recognition component (N-recognin) of the Pro/N-degron pathway; recognizes and targets for degradation proteins with N-terminal degradation signals; expressed only under starvation or osmotic stress	DNA damage stress, metal or metalloid iron stress, heat	[158, 165, 166, 167, 173]

					shock, oxidative stress, osmotic stress	
YGR144W	THI4	1.3114134	0.017717567	Thiazole synthase; abundant protein involved in the formation of the thiazole moiety of thiamine during thiamine biosynthesis; acts more as a co-substrate rather than an enzyme by providing the sulphur source for thiazole formation; undergoes a single turnover only; required for mitochondrial genome stability in response to DNA damaging agents	oxidative stress, osmotic stress	[156, 170, 174]
YOL136C	PFK27	1.2675428	0.047664313	6-phosphofructo-2-kinase; catalyzes synthesis of fructose-2,6-bisphosphate; inhibited by phosphoenolpyruvate and sn-glycerol 3-phosphate, expression induced by glucose and sucrose, transcriptional regulation involves protein kinase A	heat shock stress, osmotic stress, metal or metalloid iron stress	[160, 161, 174, 176]
YMR169C	ALD3	1.265593	0.028533969	Cytoplasmic aldehyde dehydrogenase involved in ethanol oxidation. Involved in pantothenic acid production through the conversion of 3-aminopropanal to beta-alanine, an intermediate in pantothenic acid (vitamin B5) and coenzyme A (CoA) biosynthesis. {ECO:0000269|PubMed:10407263, ECO:0000269|PubMed:12586697}.	oxidative stress, heat shock, osmotic stress	[156, 162, 170, 177]
YJR159W	SOR1	1.2503415	0.04198109	sorbitol dehydrogenase; protein sequence is 99% identical to the Sor2p sorbitol dehydrogenase; expression is induced in the presence of sorbitol or xylose	metal or metalloid ion stress, heat shock	[166, 177, 178]
YPL223C	GRE1	1.2260217	2.19839E-05	Hydrophilin essential in desiccation-rehydration process; stress induced (osmotic, ionic, oxidative, heat shock and heavy metals); regulated by the HOG pathway; GRE1 has a paralog, SIP18, that arose from the whole genome duplication	oxidative stress, heat shock, osmotic stress	[160, 170]
YGL177W	YGL177W	1.2115066	0.007194614	Dubious open reading frame; unlikely to encode a functional protein, based on available experimental and comparative sequence data	DNA damage stress, metal or metalloid iron stress	[165, 166]
YBR242W	YBR242W	1.2003203	0.04198109	5'-deoxynucleotidase involved in deoxyribonucleoside monophosphate degradation; green fluorescent protein (GFP)-fusion protein localizes to the cytoplasm and nucleus; non-essential gene; YBR242W has a paralog, YGL101W, that arose from the whole genome duplication	DNA damage stress, osmotic stress	[161, 165]
YKL221W	MCH2	1.1903057	0.000241171	Protein with similarity to mammalian monocarboxylate permeases; monocarboxylate permeases are involved in transport of monocarboxylic acids across the plasma membrane but mutant is not deficient in monocarboxylate transport	osmotic stress, metal or metalloid iron stress, oxidative stress	[165, 166]
YGR146C	ECL1	1.1855104	0.015168998	Protein of unknown function; mitochondrial-dependent role in the extension of chronological lifespan; overexpression increases oxygen consumption and respiratory activity while deletion results in reduced oxygen consumption under conditions of caloric restriction;	DNA damage stress, metal or metalloid iron stress	[159, 163, 168]

Supplementary Information Table S2. Differentially Expressed Genes

				induced by iron homeostasis transcription factor Aft2p; multicopy suppressor of temperature sensitive hsf1 mutant; induced by treatment with 8-methoxypsoralen and UVA irradiation		
YJR115W	YJR115W	1.1777071	0.026521778	Putative protein of unknown function; YJR115W has a paralog, ECM13, that arose from the whole genome duplication	DNA damage stress, metal or metalloid iron stress	[165, 175]
YKL068W-A	YKL068W-A	1.0466206	0.006810718	Putative protein of unknown function; identified by homology to Ashbya gossypii	osmotic stress, oxidative stress	[162, 180, 181]
YER091C	MET6	1.036596	0.014045424	Cobalamin-independent methionine synthase; involved in methionine biosynthesis and regeneration; requires a minimum of two glutamates on the methyltetrahydrofolate substrate, similar to bacterial metE homologs	DNA damage stress, metal or metalloid iron stress	[165, 175]
YDR297W	SUR2	1.0328353	0.00463821	Sphinganine C4-hydroxylase; catalyses the conversion of sphinganine to phytosphingosine in sphingolipid biosynthesis	osmotic stress, DNA damage stress, metal or metalloid iron stress	[159, 165, 175]
YGR155W	CYS4	1.0297836	0.000100923	Cystathionine beta-synthase; catalyzes synthesis of cystathionine from serine and homocysteine, the first committed step in cysteine biosynthesis; responsible for hydrogen sulfide generation; advances passage through START by promoting cell growth which requires catalytic activity, and reducing critical cell size independent of catalytic activity; mutations in human ortholog CBS cause homocystinuria; human CBS can complement yeast null mutant	osmotic stress, DNA damage stress, oxidative stress	[163, 165, 168]
YNL234W	YNL234W	1.0297352	0.032005909	Protein of unknown function with similarity to globins; has a functional heme-binding domain; mutant has aneuploidy tolerance; transcription induced by stress conditions; may be involved in glucose signaling or metabolism; regulated by Rgt1	DNA damage stress, metal or metalloid iron stress	[165, 175]
YGR049W	SCM4	0.9564602	0.029589581	Mitochondrial outer membrane protein of unknown function; predicted to have 4 transmembrane segments; import is mediated by Tom70p and Mim1p; interacts genetically with a cdc4 mutation; SCM4 has a paralog, ATG33, that arose from the whole genome duplication	osmotic stress, DNA damage stress, metal or metalloid iron stress, oxidative stress	[159, 163, 165, 168]
YLR299W	ECM38	0.9186672	0.042453971	Gamma-glutamyltranspeptidase; major glutathione-degrading enzyme; involved in detoxification of electrophilic xenobiotics; expression induced mainly by nitrogen starvation	osmotic stress, heat shock	[160, 161, 163, 174, 175]
YGR095C	RRP46	0.9005291	0.000904754	Exosome non-catalytic core component; involved in 3'-5' RNA processing and degradation in both the nucleus and the cytoplasm; has similarity to E. coli RNase PH and to human hRrp46p (EXOSC5)	heat shock, osmotic stress	[161, 163, 174, 176]
YMR118C	SHH3	0.8810088	0.018082702	Putative mitochondrial inner membrane protein of unknown function; although similar to paralogous Sdh3p, Shh3p is not a stoichiometric subunit of either succinate dehydrogenase or of the TIM22	osmotic stress, DNA damage stress, metal or	[161, 165, 166]

				translocase; SHH3 has a paralog, SDH3, that arose from the whole genome duplication	metalloid iron stress	
YGL178W	MPT5	0.8595471	0.031752304	mRNA-binding protein of the PUF family; binds to specific mRNAs, often in the 3' UTR; has broad specificity and binds to more than 1000 mRNAs (16% of the transcriptome); recruits the CCR4-NOT deadenylase complex to mRNAs along with Dhh1p and Dcp1p to promote deadenylation, decapping, and decay; also interacts with the Caf20p translational initiation repressor, affecting its mRNA target specificity	osmotic stress, environmental stress, DNA damage stress	[163, 165, 181]
YBL054W	TOD6	0.793665	0.034144232	PAC motif binding protein involved in rRNA and ribosome biogenesis; subunit of the RPD3L histone deacetylase complex; Myb-like HTH transcription factor; hypophosphorylated by rapamycin treatment in a Sch9p-dependent manner; activated in stochastic pulses of nuclear localization	environmental stress, DNA damage stress, oxidative stress	[165, 168, 181]
YLR044C	PDC1	0.6928362	0.029049994	Major of three pyruvate decarboxylase isozymes; key enzyme in alcoholic fermentation; decarboxylates pyruvate to acetaldehyde; involved in amino acid catabolism; subject to glucose-, ethanol-, and autoregulation; activated by phosphorylation in response to glucose levels; N-terminally propionylated in vivo; protein tyrosine nitration on Tyr157 or Tyr344 inhibits activity and impairs fermentation	ethanol stress, heat shock, osmotic stress	[160, 161, 182]
YAR018C	KIN3	-0.805308	0.032277869	Nonessential serine/threonine protein kinase; possible role in DNA damage response; influences tolerance to high levels of ethanol	osmotic stress, DNA damage stress	[163, 165, 169]
YLR190W	MMR1	-0.890892	0.026521778	Phospholipid binding protein; interacts with mitochondria and with Myo2p, functioning as an adaptor that recruits Myo2p and facilitates actin-based transport of mitochondria to the bud; mediates mitochondria anchorage at the bud tip; phosphorylated protein that localizes to the mitochondrial outer membrane; mRNA is targeted to the bud via the transport system involving She2p; member of the DSL1 family of tethering proteins	environmental stress, DNA damage stress, metal or metalloid iron stress	[159, 165, 182]
YAL023C	PMT2	-1.065132	0.000879719	Protein O-mannosyltransferase of the ER membrane; transfers mannose residues from dolichyl phosphate-D-mannose to protein serine/threonine residues; involved in ER quality control; functions as a heterodimer with Pmt2p but can also pair with Pmt5p; antifungal drug target; PMT2 has a paralog, PMT3, that arose from the whole genome duplication	osmotic stress, environmental stress, DNA damage stress	[163, 165, 181]
YMR032W	HOF1	-1.240539	0.000586242	F-BAR protein that regulates actin cytoskeleton organization; binds and bundles actin filaments, linking them to septins; required for cytokinesis, actin cable organization, and secretory vesicle trafficking; regulates actomyosin ring dynamics and septin localization; N-term. half controls cell size and actin cable levels, while the C-term. half controls actin cable organization, inhibiting Bnr1p-mediated actin nucleation; forms axial striations/pillars at the bud neck; phosphorylated by Dbf2p	ethanol stress, osmotic stress	[161, 174, 183]

Supplementary Information Table S2. Differentially Expressed Genes

YLL067C	YLL067C	-1.253496	0.018082702	Putative Y' element ATP-dependent helicase	osmotic stress, oxidative stress	[156, 161]
YOR315W	SFG1	-1.257929	0.00935186	Putative transcription factor; induces superficial pseudohyphal growth, positively regulates invasive growth, but is not required for invasive pseudohyphal growth; may act together with Phd1p; promotes cell adhesion independent of Flo11p by repressing genes that encode cell wall degrading enzymes; localizes to the nucleus; potential Cdc28p substrate	environmental stress, DNA damage stress, metal or metalloid iron stress	[165, 182]
YHR214W-A	YHR214W-A	-1.301939	0.001486807	Dubious open reading frame; induced by zinc deficiency; YHR214W-A has a paralog, YAR068W, that arose from a segmental duplication	environmental stress, metal or metalloid iron stress	[175, 182]
YOR247W	SRL1	-1.516424	0.001030188	Mannoprotein that exhibits a tight association with the cell wall; required for cell wall stability in the absence of GPI-anchored mannoproteins; has a high serine-threonine content; expression is induced in cell wall mutants; SRL1 has a paralog, SVS1, that arose from the whole genome duplication	osmotic stress, DNA damage stress	[165, 168]
YNCL0018W	RDN5	-2.087843	0.00935186	5S ribosomal RNA (5S rRNA); only complete sequence of 6 repeated RDN5 alleles; able to support viability when provided as sole form of 5S rRNA; component of the large (60S) ribosomal subunit; localized to the nucleolus via interaction with Rpl5p; may play a role in translational frame fidelity; transcription is mediated by PolIII and activated by TFIIIA and TFIIIE		
YGL263W	COS12	-2.179158	0.000586242	Endosomal protein involved in turnover of plasma membrane proteins; member of the DUP380 subfamily of conserved, often subtelomeric COS genes; required for the multivesicular vesicle body sorting pathway that internalizes plasma membrane proteins for degradation; Cos proteins provide ubiquitin in trans for nonubiquitinated cargo proteins	DNA damage stress, metal or metalloid iron stress, oxidative stress	[159, 165, 168]
YBL048W	RRT1	-2.662624	2.36686E-13	Putative protein of unknown function; conserved across S. cerevisiae strains; identified in a screen for mutants with increased levels of rDNA transcription	osmotic stress, oxidative stress, DNA damage stress	[156, 161]
YCR018C-A	YCR018C-A	-5.72767	1.88198E-54	Putative protein of unknown function; encoded opposite a Ty1 LTR	DNA damage stress, heat shock	[165, 184]
YBL049W	MOH1	-6.428332	3.13284E-58	Protein of unknown function, essential for stationary phase survival; not required for growth on nonfermentable carbon sources; possibly linked with vacuolar transport	oxidative stres, heat shock, DNA damage stress	[168, 169, 170, 185]

Supplementary Information Table S3. SGD, GO Terms

GO ID	TERM	NUM LIST ANNOTATIONS	LIST SIZE	CLUSTER FREQUENCY	TOTAL NUM ANNOTATIONS	POPULATION SIZE	GENOME FREQUENCY	ANNOTATED GENES
GO:0009058	biosynthetic process	10	45	22.22%	1069	6312	16.94%	CYS4, MPT5, PHO92, PMT2, THI4, YER091C, YGR087C, YGR095C, YLR044C, YMR169C
GO:0044281	small molecule metabolic process	6	45	13.33%	350	6312	5.54%	CYS4, SOR1, YER091C, YGR087C, YLR044C, YMR169C
GO:0006520	amino acid metabolic process	5	45	11.11%	161	6312	2.55%	CYS4, YER091C, YGR087C, YLR044C, YMR169C
GO:0009056	catabolic process	5	45	11.11%	442	6312	7.00%	ECM38, YGR066C, YGR087C, YLR044C, YMR169C
GO:0006790	sulfur compound metabolic process	4	45	8.89%	119	6312	1.89%	CYS4, ECM38, THI4, YER091C
GO:0005975	carbohydrate metabolic process	3	45	6.67%	161	6312	2.55%	SOR1, YLR044C, YOL136C
GO:0071554	cell wall organization or biogenesis	3	45	6.67%	207	6312	3.28%	HOF1, PMT2, YOR247W
GO:0034655	nucleobase-containing compound catabolic process	2	45	4.44%	46	6312	0.73%	YBR242W, YOL136C
GO:0040007	growth	1	45	2.22%	88	6312	1.39%	YOR315W

Supplementary Information Table S3. SGD, GO Terms

GO:0048646	anatomical structure formation involved in morphogenesis	1	45	2.22%	136	6312	2.15%	YJR094C
GO:0055085	transmembrane transport	1	45	2.22%	304	6312	4.82%	HXT4
GO:0007010	cytoskeleton organization	1	45	2.22%	264	6312	4.18%	HOF1
GO:0006950	response to stress	1	45	2.22%	222	6312	3.52%	PMT2
GO:0006399	tRNA metabolic process	1	45	2.22%	46	6312	0.73%	YGR095C
GO:0006091	generation of precursor metabolites and energy	1	45	2.22%	60	6312	0.95%	YLR044C
GO:0007155	cell adhesion	1	45	2.22%	22	6312	0.35%	YOR315W
GO:0006810	transport	1	45	2.22%	170	6312	2.69%	PMT2
GO:0007165	signal transduction	1	45	2.22%	368	6312	5.83%	PMT2
GO:0007034	vacuolar transport	1	45	2.22%	168	6312	2.66%	YGL263W
GO:0022607	cellular component assembly	1	45	2.22%	214	6312	3.39%	HOF1
GO:0006629	lipid metabolic process	1	45	2.22%	317	6312	5.02%	SUR2

Supplementary Information Table S4. The assignments of the IR bands

WT vs *moh1*Δ		
**Band Location (cm^{-1})**	**Band Assignment**	**References**
**2925**	CH_2 antisymmetric stretching: mainly lipids with little contribution from proteins	[186, 187]
**2857**	CH_2 symmetric stretching: mainly lipids with little contribution from proteins	[186, 187]
**1741**	Carbonyl ester stretching: lipids	[186, 187]
**1641**	Amide I: proteins (80% protein C=O stretching, 10% protein N–H bending, 10% C–N stretching)	[186, 187]
**1534**	Amide II: proteins (60% protein N–H bending, 40% C–N stretching)	[186, 187]
**1151**	β1,3 glucans	[188, 189]
**1104**	β1,3 glucans	[188, 189]
**1078**	β1,3 glucans	[188, 189]
**1042**	Mannans	[188, 189]
**991**	β1,6 glucans	[188, 189]
**917**	Mannans	[188, 189]
**805**	Mannans	[188, 189]

Supplementary Information Table 5. Primers

**Primer Name**	**Sequence (5’-3’)**
URA3 UPS_FP	CGCATCTCGAGCATTTGCATCCATACATTTTGATGGCCGC
URA3 UPS_REP	CGCATGGCTAGCGGTTGTTTATGTTCGGATGTGATGTGAGAACTG
URA3 DNS_FP	CGCATGAATTCTAATCAGTACTGACAATAAAAAGATTCTTGTTTTCAAG
URA3 DNS_REP	CGCATGGGATCCACATACGATTGACGCATGATATTA
URA3_FP	CGCATGCTAGCATGTCGAAAGCTACATATAAGGAACGTGCTGC
URA3_REP	CGCATGGAATTCGTTTTGCTGGCCGCATCTTCTC
UPS_FP	CGCATCTCGAGGACAGAACTCTGTCCTACTTTATC
UPS_REP	CGCATGCCATGGTTTCTTCTACAGTAAGATAAGCTTCT
DNS_FP	CGCATGAATTCTGATGTCTTCCTTTGTCTGCTATCTAGCACCTCT
DNS_REP	CGCATGGGATCCGGCTACTTGAAAACAACTGGAC
MOH1_FP	CGCATCTCGAGACCATGGCTAGCGGATTGCGTTACTCCATATATAT
MOH1_REP	CGCATGGGATCCTTTATTAGAATTCAGTACATTTACAAATGTTTTTC
KanR_FP	CTCGCGATAATGTCGGGCAATCA
KanR_REP	ATCCTGGTATCGGTCTGCGATTC
Outside (A)_FP	TACTGTACTTTGCTGACTTGCATTC
Outside (D)_REP	ACATAATCTTTGGGCGTATTACAAC

RT-qPCR Primers:

**Primer Name**	**Sequence (5’-3’)**
FCY1_qPCR FP	AAGTGTTCTCGGTCGTGGTC
FCY1_qPCR REP	GCATGGAGACAGCGTCGTAT
ALG9_qPCR FP	CACGGATAGTGGCTTTGGTGAACAATTAC
ALG9_qPCR FP	TATGATTATCTGGCAGCAGGAAAGAACTTGGG
MOH1_qPCR FP	GTTATTCCACTCTCAGCATCGATCGC
MOH1_qPCR REP	CACAGACTAAGTAGTCGCCAGTCAAC
ALD3_qPCR FP	CCTGGTTATGGTTCCGTTGTG
ALD3_qPCR REP	CAATACTGAGCCGCCAACCT
GRE1_qPCR FP	TCCCTACGGCGAAGAAAACC
GRE1_qPCR REP	TCGTCGTCCAACTGACCTTG
SRL1_qPCR FP	ACTACCACTTTAGCGCCCAG
SRL1_qPCR REP	CGCATTGGTAATGGTGGCTG
